# Mouse liver sinusoidal endothelial cell responses to the glucocorticoid receptor agonist dexamethasone

**DOI:** 10.3389/fphar.2024.1377136

**Published:** 2024-10-08

**Authors:** Sabin Bhandari, Ingelin Kyrrestad, Jaione Simón-Santamaría, Ruomei Li, Karolina Joanna Szafranska, Gianina Dumitriu, Javier Sánchez Romano, Bård Smedsrød, Karen Kristine Sørensen

**Affiliations:** Department of Medical Biology, UiT - The Arctic University of Norway, Tromsø, Norway

**Keywords:** liver sinusoidal endothelial cell(s), dexamethasone, glucocorticoids, tandem mass tag (TMT), proteome, fenestration, endocytosis, transcription factors

## Abstract

Liver sinusoidal endothelial cells (LSECs) which make up the fenestrated wall of the hepatic sinusoids, are active scavenger cells involved in blood waste clearance and liver immune functions. Dexamethasone is a synthetic glucocorticoid commonly used in the clinic and as cell culture supplement. However, the response is dependent on tissue, cell type, and cell state. The aim of this study was to investigate the effect of dexamethasone on primary mouse LSECs (C57BL/6J); their viability (live-dead, LDH release, caspase 3/7 assays), morphology (scanning electron microscopy), release of inflammatory markers (ELISA), and scavenging functions (endocytosis assays), and associated biological processes and pathways. We have characterized and catalogued the proteome of LSECs cultured for 1, 10, or 48 h to elucidate time-dependent and dexamethasone-specific cell responses. More than 6,000 protein IDs were quantified using tandem mass tag technology and advanced mass spectrometry (synchronous precursor selection multi-notch MS3). Enrichment analysis showed a culture-induced upregulation of stress and inflammatory markers, and a significant shift in cell metabolism already at 10 h, with enhancement of glycolysis and concomitant repression of oxidative phosphorylation. At 48 h, changes in metabolic pathways were more pronounced with dexamethasone compared to time-matched controls. Dexamethasone repressed the activation of inflammatory pathways (IFN-gamma response, TNF-alpha signaling via NF-kB, Cell adhesion molecules), and culture-induced release of interleukin-6, VCAM-1, and ICAM-1, and improved cell viability partly through inhibition of apoptosis. The mouse LSECs did not proliferate in culture. Dexamethasone treated cells showed upregulation of xanthine dehydrogenase/oxidase (Xdh), and the transcription regulator Foxo1. The drug further delayed but did not block the culture-induced loss of LSEC fenestration. The LSEC capacity for endocytosis was significantly reduced at 48 h, independent of dexamethasone, which correlated with diminished expression of several scavenger receptors and C-type lectins and altered expression of proteins in the endocytic machinery. The glucocorticoid receptor (NR3C1) was suppressed by dexamethasone at 48 h, suggesting limited effect of the drug in prolonged LSEC culture. Conclusion: The study presents a detailed overview of biological processes and pathways affected by dexamethasone in mouse LSECs *in vitro*.

## 1 Introduction

Liver sinusoidal endothelial cells (LSECs) constitute a unique endothelium considering their ultrastructure, gene expression, and physiological functions. Specialized LSEC functions include a high endocytic (“scavenger”) activity towards many blood-borne macromolecules, such as spent plasma proteins, oxidized lipoproteins, small, soluble immune complexes, nanoparticles, and waste products from matrix production and turnover (Bhandari et al., 2021; Smedsrød et al., 1990; Sørensen et al., 2012). For this purpose, the cells express a wide repertoire of scavenger receptors and other endocytosis receptors, and a well-developed endo-lysosomal apparatus for degradation of internalized ligands (Bhandari et al., 2021; Pandey et al., 2020; Bhandari et al., 2020). LSECs further have immune regulatory roles contributing to liver immune tolerance (Wohlleber and Knolle, 2016; Shetty et al., 2018). A third essential function of the cells is ultrafiltration of plasma. LSECs lack an organized basal lamina and are perforated with transcellular nanosized holes (average diameter approximately 100–200 nm in diameter), named fenestrae or fenestrations, arranged in sieve plates which ease the bidirectional traffic of lipoproteins and other molecules between blood and hepatocytes (Wisse et al., 1985; Szafranska et al., 2021). Loss of LSEC fenestrations, altered cell signaling, and/or reduced scavenging capacity are reported in aging and liver disease and are postulated to contribute to hepatic and extra-hepatic pathologies (Le Couteur et al., 2008; Fraser et al., 2012; Gracia-Sancho et al., 2021; Simon-Santamaria et al., 2010; Ito et al., 2007; Le Couteur et al., 2002; Antwi et al., 2023).

When placed in culture, important LSEC functions are rapidly changed; the cells become defenestrated, and downregulate several of their signature receptors, while upregulating genes linked to inflammation and endothelial dysfunction (Martinez et al., 2008; Li et al., 2022). We recently published the secreted and cell-associated proteome of rat LSECs by analyzing and comparing cells after 2 and 24 h in culture, reporting that the cells rapidly acquired an activated phenotype *in vitro*. The cell activation was significantly suppressed by the synthetic glucocorticoid dexamethasone (Dex), which also improved cell survival in culture (Li et al., 2022). In the present study we have examined Dex effects on the LSEC proteome in more detail, in the commonly used C57Bl/6J mouse model.

Glucocorticoids are steroid hormones released from the adrenal glands in a diurnal pattern and as a response to stress or inflammatory stimuli via activation of the hypothalamic-pituitary-adrenal axis (Whirledge and DeFranco, 2018). They are involved in many physiological processes, control glucose metabolism, and are important regulators of inflammatory responses. Dex is a potent, long-acting glucocorticoid and is widely used in the clinic as an anti-inflammatory drug, as well as in cell culture supplements to improve cell viability (Bailly-Maitre et al., 2002; Zhao et al., 2015). The drug readily permeates the cell plasma membrane and mediates its action mainly via binding to the intracellular glucocorticoid receptor (NR3C1). The Dex-receptor complex is translocated into the nucleus and acts as a transcriptional regulator (Quatrini and Ugolini, 2021). In addition, Dex can induce immediate effects in cells through non-genomic actions such as induction of phosphorylation of target kinases, increase in intracellular calcium, and alteration in the production of reactive oxygen and nitrogen species (Panettieri et al., 2019).

While the glucocorticoid receptor is widely spread in the body, the magnitude of the response to glucocorticoids varies between tissues, cell type, and cell state (Grontved et al., 2013; Franco et al., 2019). Further, Dex-induced responses cause differential effects on cell growth, cell differentiation, and functions, especially in immune cells, in a dose- and time-dependent manner (Quatrini and Ugolini, 2021). Dex influences largely on cellular metabolism and prolonged use of Dex in the clinic leads to hyperglycemia due to increased glycogenolysis and gluconeogenesis, and subsequently insulin resistance, ultimately leading to hepatic enlargement and steatosis (Tamez-Perez et al., 2015). Dex is frequently used in human and veterinary medicine and is metabolized mainly in the liver (Tomlinson et al., 1997). It is therefore important to know the detailed effect of Dex on different liver cells, including LSECs. Except for our recent report on Dex effects on the rat LSEC proteome (Li et al., 2022), few studies have assessed glucocorticoid-induced responses in these cells (Martinez et al., 1996; Melgert et al., 2000; Melgert et al., 2003; Broering et al., 2011). LSECs are the liver cells directly exposed to blood and since Dex is used both for short and long-term treatment in acute and chronic conditions, LSEC can be exposed to a wide range of plasma concentrations, and for various times.

In the present study, we have implemented a time series design (1, 10, and 48 h) for the proteomic study, and advanced mass spectrometry and workflows to gain the depth, accuracy, and precision to discern new mechanistic details of Dex-effects on mouse LSECs. We have also investigated dose- and/or time-dependent effects of Dex on LSEC fenestration (scanning electron microscopy), cell viability (live-dead, LDH release, caspase 3/7 assays), secretion of interleukin-6 and cellular adhesion molecules (ELISA), cell proliferation (BrdU incorporation ELISA), and scavenging function (endocytosis assays). Selected proteins that were differentially expressed in the proteome were further examined in qPCR and western blot experiments.

## 2 Materials and Methods

### 2.1 Animals and ethics

The proteomic experiments, ELISA assays, caspase 3/7 assays, live-dead assays, and scanning electron microscopy experiments were performed with liver cells from C57Bl/6J male mice from Charles River Laboratory (Sulzfeld, Germany), whereas the LDH release assay, endocytosis, western blot and qPCR experiments were performed with cells from C57BL/6JRj male mice from Janvier Lab (France). All mice were obtained directly from the vendors at the age of 5–6 weeks and acclimatized for at least 5 days, before being included in the experiments at the age of 6–12 weeks. The mice were group-housed (3-4 mice per cage) in filter-top mouse cages with aspen bedding (Scanbur, Norway), nesting material, houses, and aspen bricks as environmental enrichment (Datesand Ltd, UK). The mice had free access to fresh water and a standardized mouse diet and were kept under controlled conditions (21°C ± 1°C, relative humidity 55% ± 10%, and 12 h light/12 h dark cycle) in the animal research facility at UiT - The Arctic University of Norway. The experimental protocol was approved by the competent institutional authority at the UiT - The Arctic University of Norway, which is licensed by the National Animal Research Authority at the Norwegian Food Safety Authority (Mattilsynet, Approval IDs: UiT 03/19, 02/20, 24/20, 09/22, 12/23), and experiments were performed in compliance with the European Convention for the protection of Vertebrate Animals used for Experimental and Other Scientific Purposes. The experiments (i.e., liver perfusions for cell isolation) were performed postmortem, and the mice were euthanized by cervical dislocation before the start of the procedure.

### 2.2 Liver perfusion and purification of liver sinusoidal endothelial cells

Liver perfusion to obtain single cell suspensions followed the protocol in (Elvevold et al., 2022), and was performed between 9 a.m. and 11 a.m. to avoid differences in cell protein expression that could be attributed to variation in endogenous blood cortisol levels caused by the circadian rhythm. The liver was first perfused with calcium-free perfusion buffer (Smedsrød and Pertoft, 1985) to remove blood, then with perfusion buffer supplied with 0.02 mg/mL Liberase^™^ (Roche, Cat. No 05401127001) and 4.76 mM CaCl_2_. The digested liver was placed in a Petri dish with cold perfusion buffer with 1% bovine serum albumin (BSA, Applichem, Albumin Fraction V, Cat No A1391,0250), the Glisson´s capsule was removed, and the liver gently shaken to release the cells. Hepatocytes were removed by 2x differential centrifugation at 35 *g* for 2 min at 4°C, leaving the non-parenchymal liver cells (NPCs) in the supernatant. The supernatant was centrifuged at 300 *g* for 10 min at 4°C to spin down the NPCs, which were then resuspended in autoMACs rinsing solution with 0.5% BSA (Miltenyi Biotec Norden AB, Lund, Sweden). The cells were counted, spun down at 300 *g* for 10 min at 4°C, and incubated with CD146 MicroBeads (1 μL per 10^6^ cells: Miltenyi, Cat. No 130-092-007), in dilution 1:10 in MACS rinsing solution with 0.5% BSA for 15 min at 4°C in a rotator. Unbound microbeads were removed by diluting the cell suspension in 1 mL of rinsing solution followed by centrifugation at 300 *g* for 5 min. The enriched LSECs were resuspended in rinsing solution with 0.5% BSA and passed through a positive selection column on a MACS separator. The eluted cells were pelleted (300 *g* for 10 min) and resuspended in AIM-V medium (Gibco; Thermo Fisher, Waltham, MA), counted and seeded on fibronectin-coated petri dishes or tissue culture plates, and incubated at 37°C in low oxygen atmosphere (5% O_2_, 5% CO_2_ as recommended for LSECs (Martinez et al., 2008). The cultures were washed with prewarmed medium 30–40 min post-seeding and incubated further in AIM-V ± Dex (Fortecortin™, Merck) as indicated for the respective experiments. The total number of LSECs purified from 1 mouse liver was 4–12×10^6^ cells.

To validate the CD146 MACS method for purification of LSECs, a differential cell count was carried out on cultures fixed 2 h after plating and imaged by scanning electron microscopy (EM; described in section 2.3). At least 400 cells were included in the differential cell count per biological replicate (n = 4). This showed that the method of purification of LSECs produced cultures with > 95% fenestrated cells, which is the morphological hallmark of LSECs (Braet and Wisse, 2002). Close to 100% LSEC purity was observed in cultures at later time points by scanning EM.

### 2.3 Assessment of LSEC morphology by scanning electron microscopy (EM)

CD146+ LSECs were seeded on fibronectin-coated 24-well tissue culture plates at the same density as used in the proteomics study (0.3×10^6^ cells/cm^2^) and incubated in AIM-V medium for 30 min, washed, and incubated further in AIM-V ± Dex. Experiments were performed with LSEC cultures from 3 biological replicates. The cultures were incubated for 2 h (without Dex), and 24, 48, 72, or 120 h ± Dex (doses 0.1, 1.0, and 2.5 µM), then fixed in McDowell´s fixative for EM (McDowell and Trump, 1976). Medium was changed after 24 and 72 h. The fixed LSEC cultures were then stamped out from the culture plate and processed for scanning EM as previously described (Bhandari et al., 2020). The specimens were mounted on aluminum stubs, sputter-coated with gold/palladium alloy and scanned in a Zeiss Sigma Field Emission Scanning electron microscope run at 2 kV. At each time point and treatment, overview images (1,000x magnification, pixel size of 90 nm) were taken at random from at least 3 different areas per cell culture, and higher magnification images (5,000x, pixel size of 15 nm) were taken at random within these areas for validation and detailed analysis of the cell morphology.

*Semiquantitative LSEC morphology analysis*: To describe the fenestration status of the cells at different time points ± Dex, a semiquantitative analysis was performed on the scanning EM images as described (Zapotoczny et al., 2022). Each overview image contained approximately 80 cells that were manually assigned to one of four groups: 1) Highly fenestrated cells–nearly the whole cell body is fenestrated; 2) normally fenestrated cells–a wide range of regularly fenestrated cells with fenestrae arranged in sieve plates; 3) low fenestrated cells–cells with just a few sieve plates; 4) defenestrated cells–cells without fenestrations or with only few, scattered fenestrae but with intact cell body. Cells with altered morphology that suggest cell death were excluded from analysis. None of the samples contained above 10% dead cells which is a normal level for primary LSEC culture. For each sample 3-5 overview images were analysed (∼350 cells per treatment group for each biological replicate).

### 2.4 Sample preparation, TMT labelling, and peptide fractionation

**FIGURE 1:**
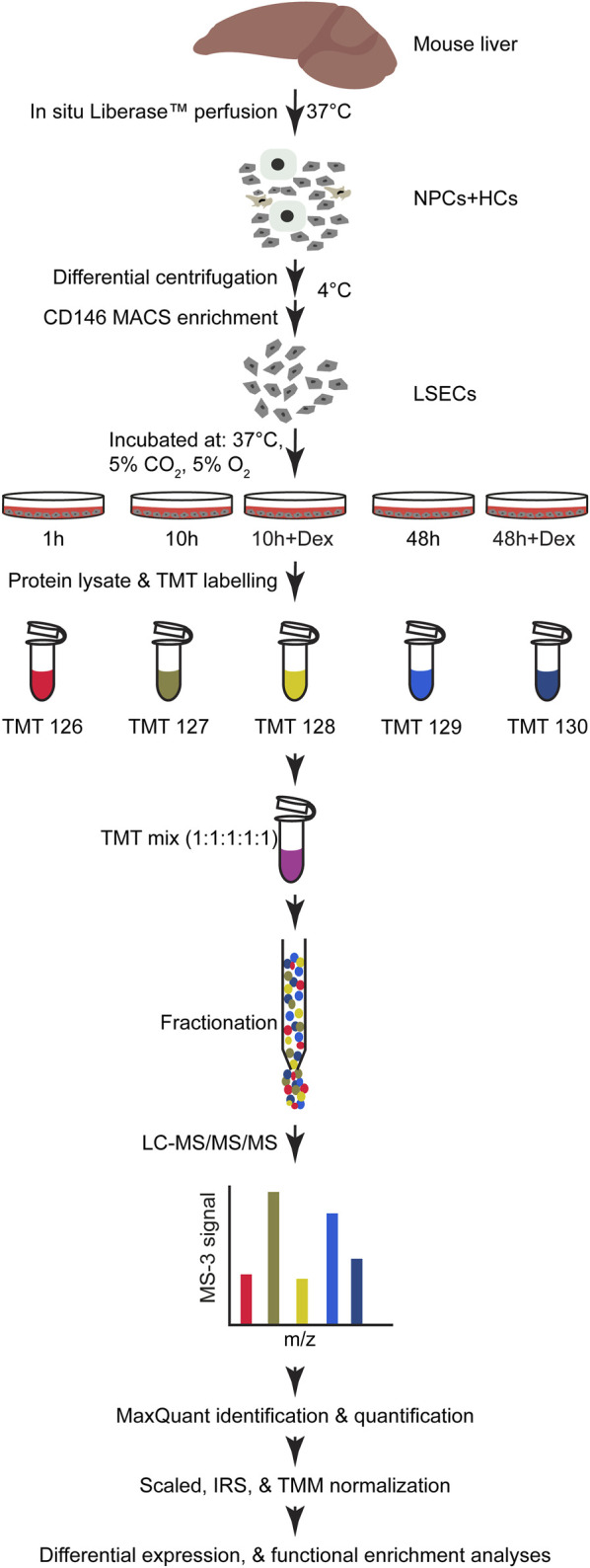
Experimental design and workflow of the proteomics experiment and analyses. Illustration of the workflow for the generation of samples for proteomic analyses from purified mouse LSECs. Liver cells were isolated by warm *in situ* liver perfusion with Liberase™, as described in Materials and Methods. The obtained single-cell suspension was then kept at 4°C during LSEC purification. LSECs were enriched from non-parenchymal liver cells on a MACS column using beads with antibodies to CD146. The cells were seeded on fibronectin-coated tissue culture plates and incubated at 37°C in 5% CO_2_ and 5% O_2_ atmosphere and allowed to attach for 30 min before washing with prewarmed medium. The plates were examined for cell density and purity and incubated with fresh medium with or without Dex for 10 and 48 h, and cells lysed to collect protein at the specified time points. The lysates were enzymatically digested to generate peptides that were labelled with TMT reporters. The samples were pre-run to determine the mixing ratio to generate the final 1:1:1:1:1 TMT mix. The labelled peptides were fractionated before LC-MS/MS/MS. The subsequent data processing is described in Materials and Methods. Biological replicates, n = 3.

The set-up for the proteomics experiment is described in Figure 1. LSECs (0.3×10^6^ cells/cm^2^) were seeded on 60 mm fibronectin-coated tissue culture plates (Sarstedt) in AIM-V medium, one plate per treatment and time point, and gently washed with prewarmed medium after 30 min. Dex treatment (1 μM = 0.4 μg/mL) started immediately thereafter. Supernatants were removed and the cell-associated proteins extracted at 1 h (LSECs in AIM-V alone), 10 h (LSECs ± Dex), or 48 h (LSECs ± Dex) post-seeding. The experiment was repeated with 3 biological replicates, each consisting of pooled LSECs from 4-5 mouse livers.

The protein extracts were prepared according to the protocol provided in the TMTsixplex Isobaric Mass Tagging Kit (Thermo Fisher, Cat No 90064) with the following modification: Denaturing reagent was 5% sodium deoxycholate in 100 mM triethylammonium bicarbonate (TEAB). Protein concentrations were measured with Direct Detect™ Infrared Spectrometer (Millipore). The proteins were then reduced according to the protocol (Thermo Fisher), except that the reducing reagent was 5 mM dithiothreitol (Sigma) instead of tri (2-carboxyethyl)-phosphine. Proteins were precipitated with acetone and the pellet was collected by centrifugation at 8,000 *g* for 10 min. The protein pellet (25 µg) was resuspended in 2 M urea and 50 mM TEAB. Proteins were digested for 6 h with 1:100 (w/w) lysyl endopeptidase (Fujifilm Wako Chemicals Europe GmBH, Neuss, Germany). The samples were diluted to 1 M urea and digested overnight with 1:20 (w/w) trypsin (V511A, Promega Corporation, WI). Peptides from each sample were then labelled with the TMTsixplex™ Isobaric Mass Tagging Kit according to the manufacturer’s protocol. OMIX C18 tips (Varian Inc., Palo Alto, CA) were used for sample cleanup and concentration.

The labelled peptides were fractionated by high pH reversed-phase chromatography (Stein et al., 2013) using an Ultimate 3,000 offline HPLC: 100 µg of peptides were reconstituted in 200 mM ammonium formate, pH 10, and loaded onto an Acuity UPLC BEH Shield RP18 (1.7 µm, 2.1 × 100 mm) column (Waters Chemistry, Milford, MA). The samples were then fractionated using a 0%–60% linear gradient of a buffer consisting of 90% acetonitrile, 20 mM ammonium formate, pH 10, at a fixed flow rate of 150 μL/min for 60 min. Forty-two fractions were collected from each TMT mix and pooled into 21 fractions using the mixing strategy fraction 1+ fraction 22, fraction 2 + fraction 23, etc. The fractions were dried in a SpeedVac concentrator (SC250, Thermo Fisher) and frozen at −80°C until MS. The samples were reconstituted in 0.1% formic acid and injected into a trap column (Acclaim PepMap 75 μm × 2 cm, C18, 3 μm, 100 Å; Thermo Fisher) for desalting before elution to the separation column (EASY-Spray column, C18, 2 μm, 100 Å, 50 μm, 50 cm; Thermo Fisher). Peptides were fractionated using a 4%–40% gradient of increasing amounts of 80% acetonitrile in water over 120 min at a flow rate of 300 nL/min. The mobile phase contained 0.1% formic acid. Samples were analyzed by an Orbitrap Fusion Lumos mass spectrometer (Thermo Fisher), using the TMT synchronous precursor selection (SPS) multi-notch MS3 quantitative method (Navarrete-Perea et al., 2018).

### 2.5 TMT data preparation and analysis

The raw files from Orbitrap Fusion Lumos were fed into MaxQuant (version 1.6.10) for processing and generation of peak lists. Peak lists were searched for identification with the MaxQuant integrated Andromeda search engine against the UniProt *Mus musculus* (mouse) reference proteome (UniProt, 2021) with the following parameters: 2 missed cleavages were allowed at max; carbamidomethyl and TMT labelling (at N-terminus and lysine residue) were set as fixed modification, while oxidation at methionine and acetylation at the protein N-terminus were set as variable modifications. The mass tolerance was set to 4.5 ppm and 20 ppm, respectively, for the precursor ions and the fragment ions. None of the peaks were excluded for any known contaminants. A false discovery rate (FDR) of 1% was applied to eliminate false positives at both peptide and protein level.

The protein groups output text file from the MaxQuant was uploaded into Perseus, version 1.6.14.0 (Tyanova et al., 2016) for initial data processing to filter out irrelevant protein groups with identification tag “Only identified by site”, “Reverse” and “Potential contaminants”. The annotation of the protein IDs to their corresponding gene symbols was manually curated with the UniProt Knowledgebase (UniProt, 2021) using Retrieve/ID mapping. The tag-reporter intensity corrected from MaxQuant was used for protein quantification. The intensity corresponding to redundant gene symbols associated with a protein group was summed before differential expression analysis. Each TMT run had all five samples from a biological replicate; see experimental set up in Figure 1. The factor for global scaling normalization was determined separately for each run. Subsequently, after scaling the dataset, internal reference scaling normalization (Plubell et al., 2017) was used to correct the effect of the different TMT runs. Finally, the compositional bias was corrected using TMM normalization (Robinson and Oshlack, 2010) and tested for differential expression with edgeR (3.30.0) (Robinson et al., 2010).

### 2.6 Live/dead assay

LSEC cultures were established in fibronectin-coated 48-well tissue culture plates (Sarstedt; 0.25 × 10^6^ cells/well), washed after 40 min, and incubated further in AIM-V medium ± Dex (1, 10, 100, 1,000 μM) for 2 h (only without Dex), 24, 48, or 72 h, at 37°C in 5% O_2_, 5% CO_2_. Separate plates were used for each timepoint. Cell viability was assessed with Invitrogen Live/Dead™ Cell Imaging Kit (488/570, Cat. No R37601). The reagent was present during the last 15 min of the incubation time and cells were imaged in a widefield microscope (Zeiss Cell Discoverer 7). Ten images were taken automatically with ×10 magnification at 10 preset locations within each well, and the live (green) and dead (red) cells were separated and counted automatically with the software Cell Profiler (copyright Broad Institute). The average number of cells per image was approximately 2,500 in the 2 h control cultures. The experiment was repeated with three biological replicates (n = 3), each done with two technical replicates.

### 2.7 Lactate dehydrogenase (LDH) release assay

The cytotoxic effect of Dex was analyzed with the LDH-Glo Cytotoxic assay (Promega, Cat. No J2380). LSEC cultures were established in fibronectin-coated 24-well tissue culture plates (Sarstedt, 6×10^5^ cells/well), washed after 40 min, and incubated in 0.5 mL AIM-V medium ± Dex (1, 10, 100, 1,000 μM) at 37 °C in 5% O_2_, 5% CO_2_ for up to 48 h. At 2, 24, and 48 h, 25 μL of the medium was collected and frozen at −20 °C in LDH storage buffer, until analysis. At each time point, parallel cultures were dissolved in Triton X-100 (final concentration 0.1%) and used as a positive control. Luminescence was detected at emission 540–550 nm in a CLARIOstar Plus microplate reader (BMG Labtech GMbH, Ortenberg, Germany).

### 2.8 Cell proliferation assay

Proliferation of LSECs in culture was assessed with a BrdU incorporation assay (ELISA kit, Abcam, Cat. No ab126556). LSEC cultures were established in fibronectin-coated 96-well plates (Corning Costar^®^ 3,903; 1×10^5^ cells/well). Mouse embryonic fibroblasts (MEF; SC1 CRL-1404, ATCC, US) were used as positive control and plated in uncoated wells at a density of 1.5×10^4^ cells per well. LSECs were replenished with fresh AIM-V medium ± Dex (0.1, 1, 10 μM) 30 min post seeding, and incubated at 37°C, in 5% CO_2_ and 5% O_2_ for 72 h with one medium change (±Dex) after 24 h. At 72 h, cultures were replenished with fresh medium with BrdU reagent and incubated for another 24 h, then fixed for 30 min. Fixed cells were stained with anti-BrdU antibody for 1 h, then with peroxidase goat anti-mouse IgG conjugate for 30 min, before TMB peroxidase substrate (100 μL/well) was added, and absorbance measured in a CLARIOstar Plus microplate reader. The experiment was repeated with three biological replicates (n = 3), each done with two technical replicates.

### 2.9 Caspase 3/7 assay

Caspase activity was assessed with Caspase-Glo^®^ 3/7 (Promega Corporation, Cat. No G8090). LSEC cultures established in fibronectin-coated, white, clear bottom 96-well plates (Corning Costar^®^ 3,903; 1×10^5^ cells/well), were replenished with 100 µL of AIM-V medium alone, or AIM-V with 1 µM Dex ± hamster anti-mouse CD95 (BD Pharmingen™, BD Biosciences, CA, Cat. No 554255; 10 ng/mL), then incubated for 2 or 24 h. The biological replicates (n = 3), each in duplicate, were run in the same plate, with one plate for each time point, and luminescence measured in a CLARIOstar Plus microplate reader.

### 2.10 Enzyme-linked immunosorbent assays for IL-6, VCAM-1, and ICAM-1

DuoSet ELISA kits for interleukin-6 (IL-6; Cat. No DY406), soluble intercellular adhesion molecule-1 (ICAM-1, Cat. No DY796), and vascular cell adhesion molecule-1 (VCAM-1, Cat. No DY643) were from R&D systems (Bio-Techne Corporation, MN). LSEC cultures were established in fibronectin-coated 24-well plates (Sarstedt; 6×10^5^ cells/well), washed after 30 min, and replenished with fresh AIM-V medium ± Dex (doses and time points are indicated in the figures). For each assay, the experiment was repeated with three biological replicates (n = 3), each done with two technical replicates. The raw optical density readouts (from CLARIOstar Plus) were used for four parameters logistic regression to determine the concentration based on the standards.

### 2.11 Endocytosis assays

Ligand labeling: Formaldehyde-treated bovine serum albumin (FSA) was prepared as described (Eskild and Berg, 1984), and labeled with carrier-free Na^125^I using Iodogen as oxidizing agent (Pierce Chemicals, Rockford, IL). The radiolabeled ligand was separated from unbound ^125^I on a PD-10 column (GE Health, Uppsala, Sweden); specific radioactivity was approximately 1-2x10^6^ counts per minute (cpm) per µg protein.

Endocytosis assays: LSEC cultures were established in fibronectin-coated 48-well tissue culture plates (Sarstedt; 0.3×10^6^ cells/well), washed after 30 min, and cultures incubated for various periods in AIM-V ± Dex, before the endocytosis experiments were started. Two series of endocytosis experiments were performed:

In experimental series 1 (biological replicates: n = 3, each done in duplicate), the LSEC uptake of trace amounts of ^125^I-FSA during a 2 h incubation period was measured in cells that had been cultured for 2, 24, 48, 72, or 120 h ± Dex (0.1, 1.0, 2.5 µM), at 37°C in 5% O_2_, 5% CO_2_, before the start of the experiment. The medium was removed, and 100 µL of AIM-V with 1% human serum albumin and ^125^I-FSA (approximately 0.1 μg/mL) were added to each culture. The cultures were incubated with the ligand for 2 h at 37 °C in 5% O_2_, 5% CO_2_, before endocytosis was measured (Li et al., 2022; Hansen et al., 2002).

In experimental series 2 (biological replicates: n = 3, each done in duplicate or triplicate), the LSEC capacity of endocytosis of FSA was measured in cells that had been cultured for 48 h in AIM-V ± 1 µM Dex, and results compared to the endocytic capacity of freshly plated LSECs. All cultures were incubated for 2 h with 100 µL of AIM-V with 1% human serum albumin and ^125^I-FSA (approximately 0.1 μg/mL) plus 0, 10, 20, 40, or 80 μg/mL of non-labeled FSA. The amount of ligand per cell was estimated based on the cell numbers in parallel cultures: For each biological replicate, parallel cultures were seeded at similar cell density, washed at the same time points, and fixed at the end of the endocytosis experiments for estimation of cell numbers per culture. Cell nuclei were stained with DAPI (Sigma-Aldrich) and counted in Axio Observer (Carl Zeiss).

In both experimental series, ligand uptake in the LSEC cultures was calculated as described (Hansen et al., 2002). In short, at the end of the incubation period, the cell supernatant was removed, the cells washed in cold PBS, and then lysed in 1% sodium dodecyl sulphate. Intact protein in the supernatant, including ^125^I-FSA that had not been endocytosed were pelleted with 20% trichloroacetic acid, whereas acid-soluble ^125^I released from the cells after lysosomal degradation of endocytosed ligand (Hellevik et al., 1996) were measured in the remaining supernatant. Radioactivity in the cell lysate and supernatant (precipitated, and acid-soluble fractions) were measured in an automated gamma counter (Cobra II, Packard). Total endocytosis was calculated as the sum of radioactivity in the cell lysate and the acid-soluble fraction of the supernatant, after adjusting for the percentage of free ^125^I in cell-free control wells and presented as percent of total ligand radioactivity added to the cultures.

### 2.12 Quantitative PCR

LSEC cultures were established in fibronectin-coated 48-well tissue culture plates (0.25 × 10^6^ cells/well) and incubated ± 1 µM Dex for 48 h. Cellular RNA was isolated and reverse transcribed into cDNA using the TaqMan™ Fast Advanced Cells-to-CT™ Kit (Thermo Fisher, Cat. No A35374) according to the manufacturer’s instructions. The cDNA was subsequently pre-amplified using TaqMan™ PreAmp Master Mix (Thermo Fisher, Cat. No 4391128) under the recommended conditions.

Quantitative PCR (qPCR) was conducted using the TaqMan™ Gene Expression Assay on a QuantStudio™ 5 Real-Time PCR System (Applied Biosystems). Assay IDs for the target genes: Foxo1, Mm00490671_m1; Xdh, MM00442110_m1; Nos2, MM00440502_m1; B2m, Mm00437762_m1. The qPCR reactions were set up in a total volume of 10 μL, containing 2.5 µL cDNA and 0.5 µL of TaqMan™ Gene Expression Assay and 5 µL of TaqMan™ Fast Advanced Master Mix for qPCR (Thermo Fisher, Cat. No 4444556). The cycling conditions were: UDG activation at 50°C for 2 min and an initial denaturation at 95°C for 20 s, followed by 40 cycles of 95°C for 1 s and 60°C for 20 s. Efficiency of primers, and copies of the target cDNA was tested and quantified with standard curves generated using gBlocks (Integrated DNA Technologies). The range of the standard curve was from 10 to 1,000,000 copies. All assays had efficiencies over 92%. The relative expression levels were calculated using the ΔΔCt method using B2m as reference gene.

### 2.13 Western blot

LSEC cultures established in 6 well plates (2.5 × 10^6^ cells/well) were incubated for 48 h ± 1 µM Dex, then lysed in RIPA buffer (Thermo Fisher, Cat No 89900) with protease inhibitor (Thermo Fisher: Cat No A32955), with N-ethylmaleimide (Thermo Fisher, Cat No 23030, and vanadate (Sigma, Cat No S6508). The cell lysate was sonicated, reduced, and heated at 70°C for 10 min. Four μg of total protein (measured by Direct Detect ®spectrometer) per sample was analyzed on SDS-Page with NuPage 4%–12% Bis-Tris gels (Invitrogen) according to manufacturer’s protocol with protein ladders Precision Plus protein Dual color standards (Bio-Rad, Cat No 1610374) and MagicMark western protein standard (Invitrogen, Cat no LC5603). Immunoblotting was done onto 0.45 µm PVDF transfer membranes (Thermo Fisher, Cat No 88518). Unspecific signal was blocked in 5% low fat powder milk in TBS with 0.1% Tween 20 (1 h, RT), incubated with primary antibody overnight, at 4°C, and with secondary antibody for 1 h, at RT. Primary antibodies were VE-cadherin rabbit polyclonal antibody (Thermo Fisher, Cat. No 36-1900, 1 μg/mL), Claudin 5 rabbit polyclonal antibody (Thermo Fisher, Cat No 34-1600, 0.25 μg/mL), JAM-A (CD321) goat polyclonal antibody (Thermo Fisher, Cat No PA5-47059, 1 μg/mL), Nectin-2 recombinant rabbit monoclonal antibody (Thermo Fisher, Cat No MA5-35822, 0.4 μg/mL). Anti-beta Actin rabbit polyclonal antibody (Abcam, Cat No ab8227, 0.2 μg/mL) was used as loading control. Secondary antibodies were Donkey anti-Goat (H + L), cross absorbed, HRP (Invitrogen, Cat No A16005, 1:10000 dilution), Goat anti rabbit IgG H&L, HRP (Abcam, Cat No ab205718, 1:30000 dilution). Some of the western blot membranes were reused for loading control. Stripping was performed in a mild stripping buffer, pH 2.2; 15 g glycine, 1 g SDS, 10 mL Tween 20 in distilled water, per L. Labeled proteins were visualized with SuperSignal West Pico Plus chemiluminescent substrate (Thermo Fisher, Cat No 34580) in ImageQuant LAS4000. ImageJ was used for relative quantification of the protein bands obtained on the membrane.

### 2.14 Statistical analysis and visualization

Preprocessing, annotation, curation, and filtrations of the proteomics data were done in the Perseus environment, ver. 1.6.14.0, (Tyanova et al., 2016). The R/Bioconductor environment (https://bioconductor.org) was used to normalize the TMT data, and the edgeR integrated exact test (Robinson and Smyth, 2008) was implemented to identify differential protein expression. Proteins that had a log2 fold change (log2FC) ≥ 0.5, and FDR ≤ 0.05 between the comparisons were deemed significantly different. The gene sets with FDR q-value ≤ 0.05 obtained from the gene set enrichment analysis (GSEA) were identified as significantly enriched.

Statistical analyses of data from functional experiments, cytotoxicity/viability assays, ELISA experiments, and image analyses were done in SPSS (IBM), and R, and the tests are specified in figure legends. Statistical analyses of the qPCR data were performed with GraphPad Prism (GraphPad Software). The analyses were done on biological replicates. Where several technical replicates had been included in the assay, only the average value were included in the statistical analysis, representing one biological replicate. Shapiro-Wilk test was used to assess the normality of data distribution and Levene’s test was used to evaluate homogeneity of variances.

Figures were generated using R packages including factoextra, ggplot2, ggpubr, pheatmap, and the plugins EnrichmentMap and String from Cytoscape, Microsoft Office Excel, and Adobe Illustrator.

### 2.15 Proteomics data availability

The mass spectrometry proteomics data have been deposited in the ProteomeXChange Consortium via the PRIDE (Perez-Riverol et al., 2019) partner repository with the dataset identifier PXD041381. The whole processed proteome, and comparison of protein expression between groups are included in Supplementary Material (Supplementary Table S1).

## 3 Results

### 3.1 Dose- and time-dependent effects of dexamethasone on mouse LSEC viability and morphology *in vitro*

LSECs are well-known to rapidly change their phenotype and lose viability in culture (Martinez et al., 2008; Li et al., 2022). We therefore tested the dose- and time-dependent effects of Dex on LSEC viability, cytokine production, and morphology in a series of experiments. The results were used as foundation for the design of the proteomics experiments.

**FIGURE 2:**
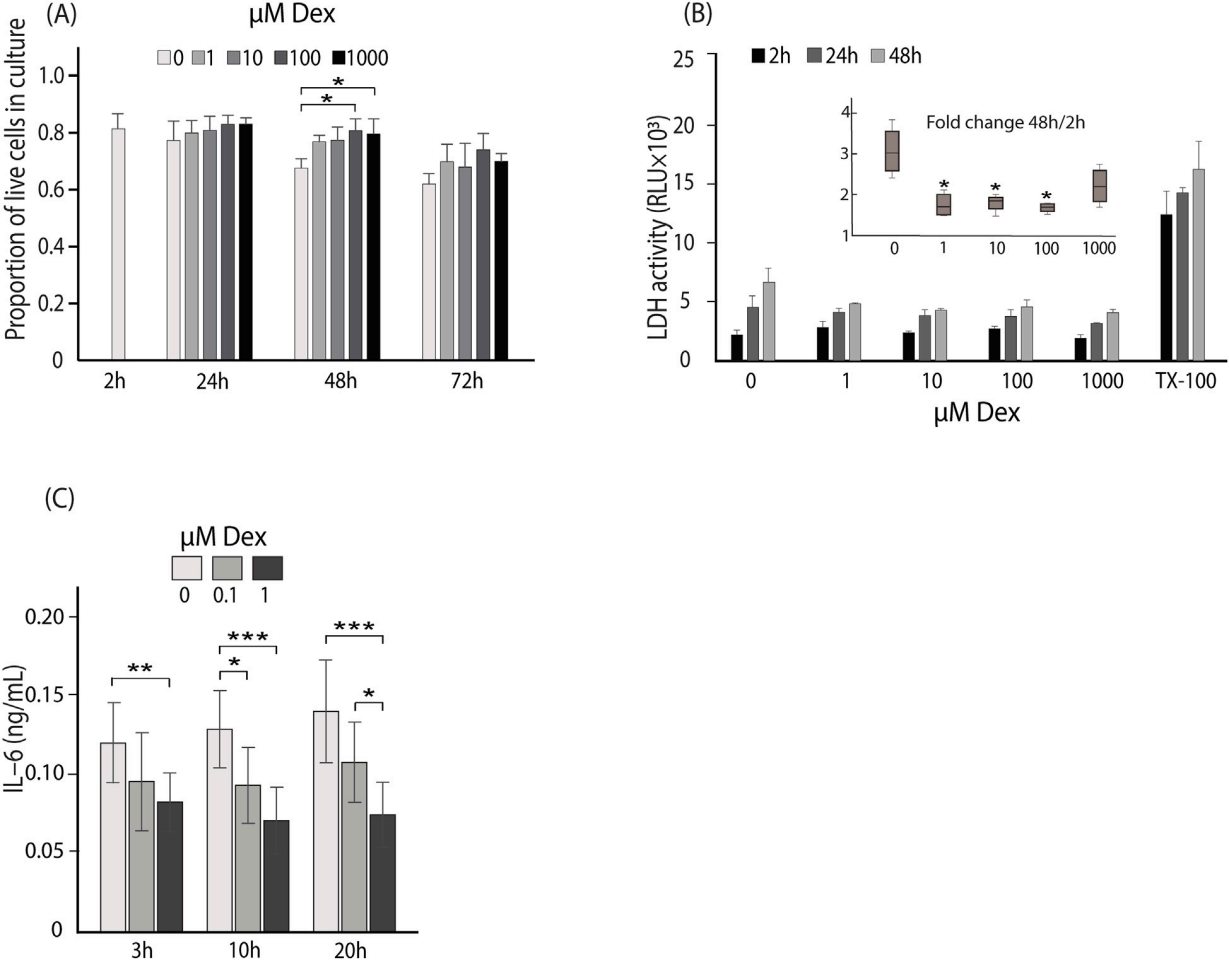
Dose and time-dependent effects of Dex on mouse LSEC viability and IL-6 production. **(A)** Live/dead assay: The bar chart shows the proportion of live cells (±SD) at 2–72 h in LSEC cultures ± Dex (0–1,000 μM). The results are mean values of 3 biological replicates ± SD. *p-value < 0.05 (One way ANOVA, with Tukey´s *post hoc* test). **(B)** The bar chart shows the LDH activity in LSEC culture supernatants. LDH activity was measured in repeated samples at 2, 24, or 48 h ± Dex (0–1,000 μM). Parallel cultures were treated with Triton X-100 (TX-100) and LDH activity measured in the whole lysate at 2, 24, or 48 h. Results are mean values of 4 biological replicates ± SD. The insert box plot shows the corresponding fold-change in LDH activity from 2 to 48 h. Median values are presented as horizonal lines, and whiskers represent interquartile range. *p-value < 0.05 (Independent-samples median test, with Bonferroni correction). **(C)** The bar chart shows the concentration of IL-6 in LSEC culture supernatants at 3, 10, and 20 h ± Dex (0, 0.1, or 1.0 μM) measured by end-point ELISA. Results represent the mean values of 3 biological replicates ± SD. *p-value < 0.05, **p-value < 0.01; ***p-value < 0.001 (ANOVA for dependent data, with Tukey´s *post hoc* test was used for comparison of data at each end point).

Dex effect on LSEC survival in culture was examined by quantifying live and dead cells after 2, 24, 48, or 72 h (n = 3), while a cytotoxic effect of Dex was examined by repeated measurements of LDH levels in supernatants of cells incubated for 48 h (n = 4). LDH release from cells is used as a marker of cytotoxicity caused by loss of plasma membrane integrity. In both assays, freshly established LSEC cultures were incubated with 0, 1, 10, 100, or 1,000 μM Dex (Figures 2A, B). The live/dead assay showed that the average ratio of viable, adherent cells was higher in cultures with Dex than without Dex at 24–72 h (significant at 48 h for 100 and 1,000 μM; Figure 2A). This trend was supported by the results of the LDH release assay. LDH release to supernatants increased with time in all cultures (Figure 2B) but the fold change between 2 and 48 h was significantly lower in the cultures with Dex for the doses 1, 10 and 100 μM Dex, than in cultures without Dex (insert in Figure 2B). This indicates that Dex has a positive effect on preserving LSEC membrane integrity and thus viability in culture. The lowest dose (1 μM) was used in the next set of experiments where we measured the effect of Dex on cytokine production and cell ultrastructure.

LSECs are major producers of IL-6 in the liver and secrete this cytokine also in early primary culture (Li et al., 2022). The effect of Dex (0.1 and 1 μM) on IL-6 secretion was therefore tested after 3, 10, and 20 h (n = 3). Dex significantly suppressed IL-6 production, with 1 μM being more effective than 0.1 μM (Figure 2C).

**FIGURE 3:**
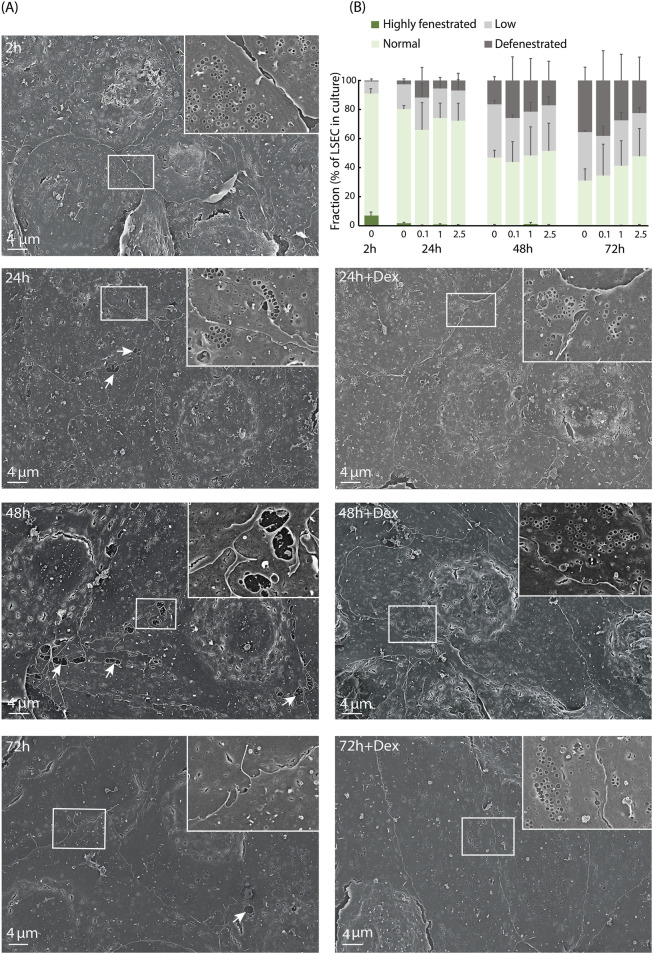
Dex effects on mouse LSEC fine structure in culture. **(A)** Scanning electron micrographs of freshly isolated mouse LSECs cultured for 2–72 h in AIM-V medium ± 1 μM Dex. Inserts show indicated details of cell borders, and arrows point to gaps between cells. **(B)** Semi-quantitative measurements of fenestration level in mouse LSECs cultured from 2 h to 72 h in AIM-V medium ± 0.1, 1.0, or 2.5 μM Dex. The cells were sorted into one of the following four categories: Highly, normal, and lowly fenestrated, or defenestrated as defined in Methods. Results are mean value of 3 biological replicates. In total about 350 cells were analyzed per treatment group and time point per biological replicate.

A morphological hallmark of LSECs is the numerous open pores, or fenestrae, which are normally organized in sieve plates (Wisse et al., 1985; Szafranska et al., 2021). However, this feature is rapidly lost in culture (Martinez et al., 2008). The size of individual fenestrae is below the resolution limit of the light microscope, and we therefore performed scanning EM to examine the fine structure of the cell morphology. Fenestration of LSECs (biological replicates: n = 3) following incubation with 0, 0.1, 1, or 2.5 μM Dex for 24, 48, 72, or 120 h, was compared to freshly prepared (2 h) non-treated cultures. The highest Dex dose (2.5 μM) was included in these experiments as this dose was used in a recent study of the rat LSEC proteome (Li et al., 2022). LSECs established at a density of 0.3×10^6^ per cm^2^ in serum-free AIM-V medium formed a continuous monolayer for at least 5 days both in the presence and absence of Dex, with the highest cell density observed with Dex (Supplementary Figure S1A). A general observation was that LSECs cultured in the presence of Dex made closer contact between cells, and had smoother cell borders, compared to the time-matched non-treated cells. This was most evident at 48–72 h after isolation (Figure 3A).

In all cultures, the LSECs lost their fenestrae over time. At 2 h, more than 95% of the cells were fenestrated, proving their identity as LSECs (Figures 3A, B). At 24 h non-treated cultures showed slightly more fenestrated cells than the Dex-treated cultures (not significant). However, defenestration occurred at a slower rate in cultures with Dex (Figure 3B, not significant), and after 5 days, some cells with sieve plates were still observed in the Dex-treated cultures whereas cultures without Dex were almost totally defenestrated (Supplementary Figure S1B).

To check if the cells after 5 days represented defenestrated LSECs or represented the proliferation of an initially small number of contaminating non-fenestrated endothelial cells, we performed a cell proliferation assay (BrdU incorporation). The results showed no cell proliferation in the LSEC cultures with or without Dex supplementation (Supplementary Figure S1C).

In summary, we observed the trend that Dex in the doses tested (1–1,000 μM) improved LSEC survival in culture, with the best effect observed at 48 h. Dex further repressed culture-induced IL-6 secretion in LSECs already after 3–10 h, and best with 1 μM of the two doses tested (0.1, 1 μM). Dex in this dose also showed positive effect on LSEC morphology. Based on these observations, we went for a time-course design in the quantitative proteomics experiments to determine 1) early effects of Dex, from 1–10 h, and 2) later effects, from 10–48 h, using 1 μM as this dose was in the lower range of the tested doses but still considered to show an effect on the proteome.

### 3.2 Global analysis revealed a rapid change in the LSEC proteome *in vitro*, which was slightly modified by Dex

The general workflow of the TMT quantitative proteomic study is illustrated in Figure 1. Proteins were collected from LSEC cultures at 1, 10, and 48 h, and the experiment was repeated with 3 biological replicates. 6,028 non-redundant protein IDs were identified, quantified, and used in the subsequent downstream analyses. The whole processed proteome and comparison of proteins between groups are shown in Supplementary Table S1.

**FIGURE 4:**
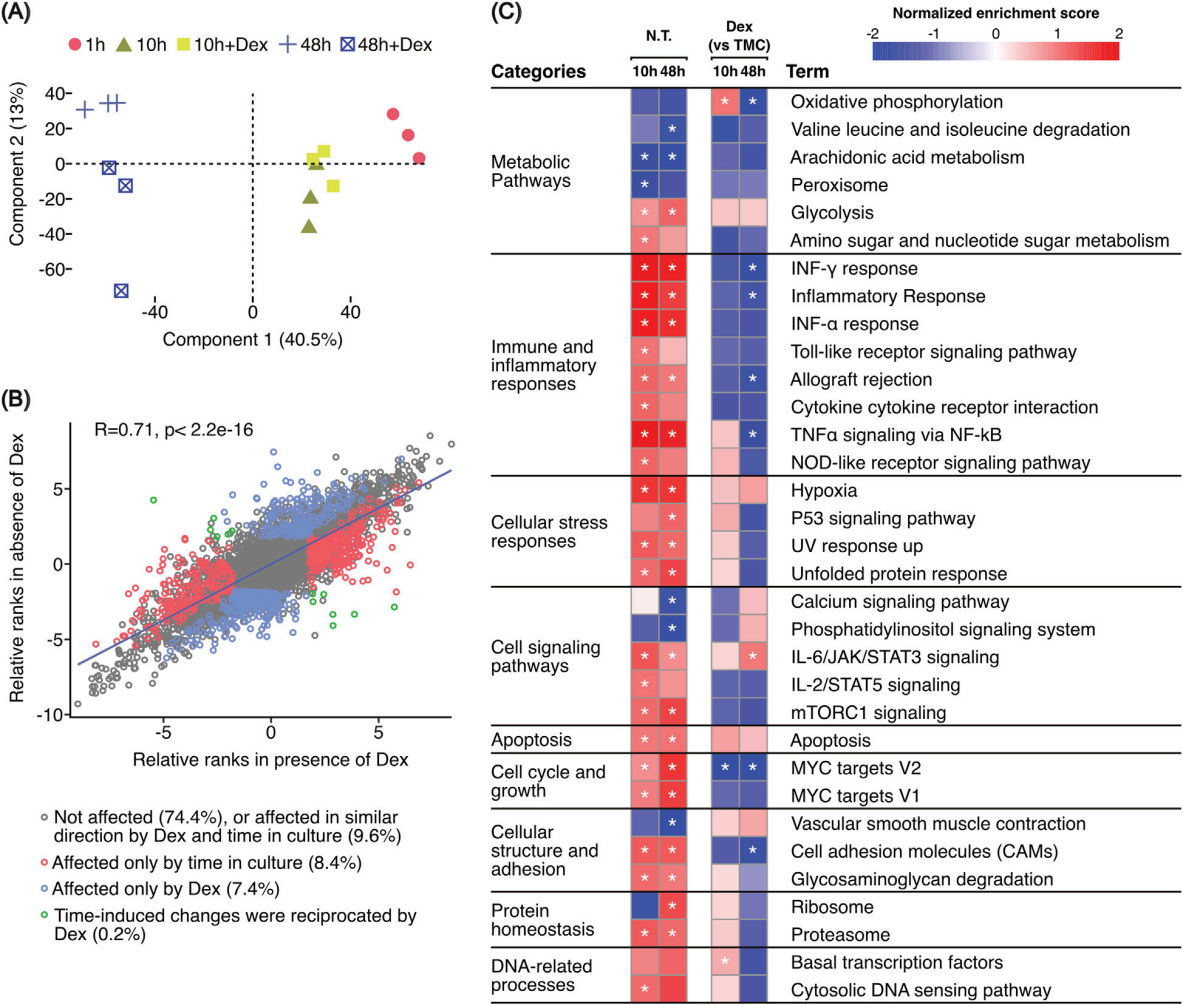
Global aspects of the proteomics data. **(A)** Principal component analysis (PCA) plot based on scaled TMT reporter intensities of all samples included in the proteomics experiment. **(B)** Scatter plot illustrating Dex-correlated changes in the LSEC proteome on top of changes correlated with culture-time. This was done by plotting the ranks, calculated from the generalized linear model with edgeR, of [(48 h vs. 10 h) vs. (10 h vs. 1 h)] against [(48 h + Dex vs. 10 h + Dex) vs. (10 h + Dex vs. 1 h)]. **(C)** Scaled heatmap illustrating the results of the GSEA analysis based on hallmark gene sets and KEGG legacy pathway gene sets defined in MSigDB (ver.7.2) (Subramanian et al., 2005). Only gene sets with FDR q ≤ 0.05 were identified as enriched (marked with a star). We used the Signal2Noise parameter for ranking genes and weighted options for enrichment statistics during GSEA. The color code is based on the normalized enrichment score (NES) obtained from the GSEA analysis. The underlying data for the figure is in Supplementary Table S2. N.T, not treated with dexamethasone (Dex). TMC, time-matched control.

Principal component analysis (Figure 4A) showed a large separation between samples over the three timepoints indicating a substantial effect of time in culture on the composition of the LSEC proteome (component 1). At 10 h Dex-treated and non-treated samples were partly overlapping, while at 48 h Dex- and non-treated samples were segregated into two separate clusters (component 2).

To understand in more detail how the protein expression changed over time and in response to Dex, we compared the ranks of protein expression between different timepoints and treatments. We found a moderately high correlation (R = 0.71, *p* < 2.2e-16) between the ranks obtained from pairwise comparison of the non-treated samples [(48 h vs. 1 h) vs. (10 h vs. 1 h)], and the Dex-treated samples [(48 h + Dex vs. 1 h) vs. (10 h + Dex vs. 1 h)] using a generalized linear model from edgeR (scatter plot in Figure 4B). This showed that the expression of most proteins (74.4%) was unaffected by time in culture, 9.6% was affected in a similar direction with time both with and without Dex, 8.4% was affected in a time-dependent manner only, and 7.4% in a Dex-dependent manner only. In a small number of proteins (0.2%), Dex reciprocated the time-induced changes *in vitro*.

Taken together, these analyses indicate a significant effect of culture time on the LSEC proteome, which is further modified by Dex treatment.

### 3.3 A major shift in metabolism and immune pathways was observed already after 10 h

Gene Set Enrichment Analysis (GSEA) was performed on the whole proteomic dataset using normalized TMT reporter intensities. The analysis was based on hallmark gene sets and KEGG legacy pathway gene sets defined in the Molecular Signatures Database (MSigDB ver 7.2) (Subramanian et al., 2005). We focused on gene sets overrepresented in at least one of four pairwise comparisons. To investigate culture-induced changes we have compared 1) non-treated samples at 10 h vs. 1 h, and 2) non-treated samples at 48 h vs. 1 h. To reveal changes induced by Dex we compared 1) samples treated for 10 h with Dex vs. 10 h without Dex, and 2) 48 h with Dex vs. 48 h without Dex (Figure 4C, Supplementary Table S2). Terms that had FDR value ≤ 0.05 were considered significant.

GSEA results revealed significant shifts in various biological processes highlighting the complex interplay of metabolic, stress response, and signaling pathways involved in LSEC adaptation to culture conditions. We observed an increase in glycolysis scores coupled with a decrease in oxidative phosphorylation scores compared to freshly plated cells (1 h control) (Figure 4C, left columns). Furthermore, we detected alterations in pathways related to cellular stress responses (hypoxia, unfolded protein response, p53 signaling, UV response up) and immune and inflammatory responses (TNFα signaling via NF-κB, interferon responses, cytokine cytokine receptor interaction, Toll-like receptor signaling pathway, Nod-like receptor signaling pathway). These changes indicate an activated LSEC phenotype in response to the new *in vitro* environment. Additionally, enrichment in pathways related to cellular structure and adhesion, cell signaling pathways, MYC targets, and apoptosis suggests that LSECs are adjusting their growth, survival mechanisms, and morphology to adapt to culture conditions (Figure 4C, left columns).

Dex treatment demonstrated a modulating effect on these pathways and processes (Figure 4C, right columns), particularly on metabolic, immune and inflammatory responses, cell adhesion molecules, and basal transcription factors. Interestingly, the GSEA revealed a biphasic response for several processes and pathways with Dex. This was most pronounced for oxidative phosphorylation, and TNFα signaling via NF-κB which both showed higher enrichment scores at 10 h and significantly lower scores at 48 h with Dex compared to non-treated time-matched controls. At 48 h, interferon-γ response, inflammatory response, allograft rejection, MYC targets, and cell adhesion molecules (CAMs) also showed significantly lower scores with Dex.

**FIGURE 5:**
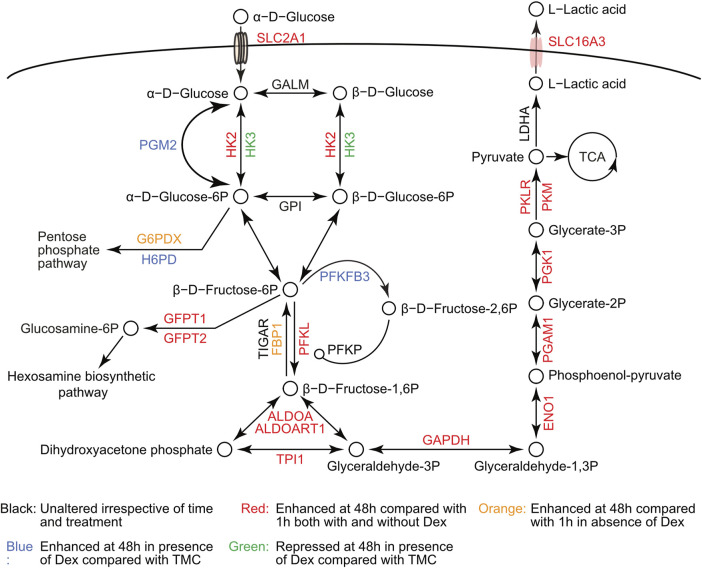
Protein changes in the glycolytic pathway during LSEC *in vitro* maintenance. Schematic drawing of the glycolytic pathway based on the KEGG pathway illustration, illustrating changes in protein level as a function of time in culture and Dex treatment. The underlying data for the figure is in Supplementary Table S3. Unfilled circles represent the glycolytic metabolites. Arrowheads show the direction of the reactions driven by the glycolytic enzymes. The small circle (o-) between PFKP and PFKL represents allosteric enhancement.

The proteomics data indicated an increased glycolytic flux in LSECs when placed in culture, as illustrated in Figure 5 (supporting data in Supplementary Table S3). While most glycolytic proteins were enhanced with time in culture both with and without Dex, some were significantly higher expressed with Dex compared to time-matched controls. These included phosphoglucomutase-2 (PGM2), 6-phosphofructo-2-kinase/fructose-2,6-biphosphatase 3 (PFKFB3), which is an allosteric regulator of phosphofructokinase, and hexose-6-phosphate dehydrogenase (H6PD). On the contrary, Dex repressed the expression of hexokinase 3 (HK3) but not HK2.

### 3.4 Culture-induced activation of LSECs was partly suppressed by Dex

**FIGURE 6:**
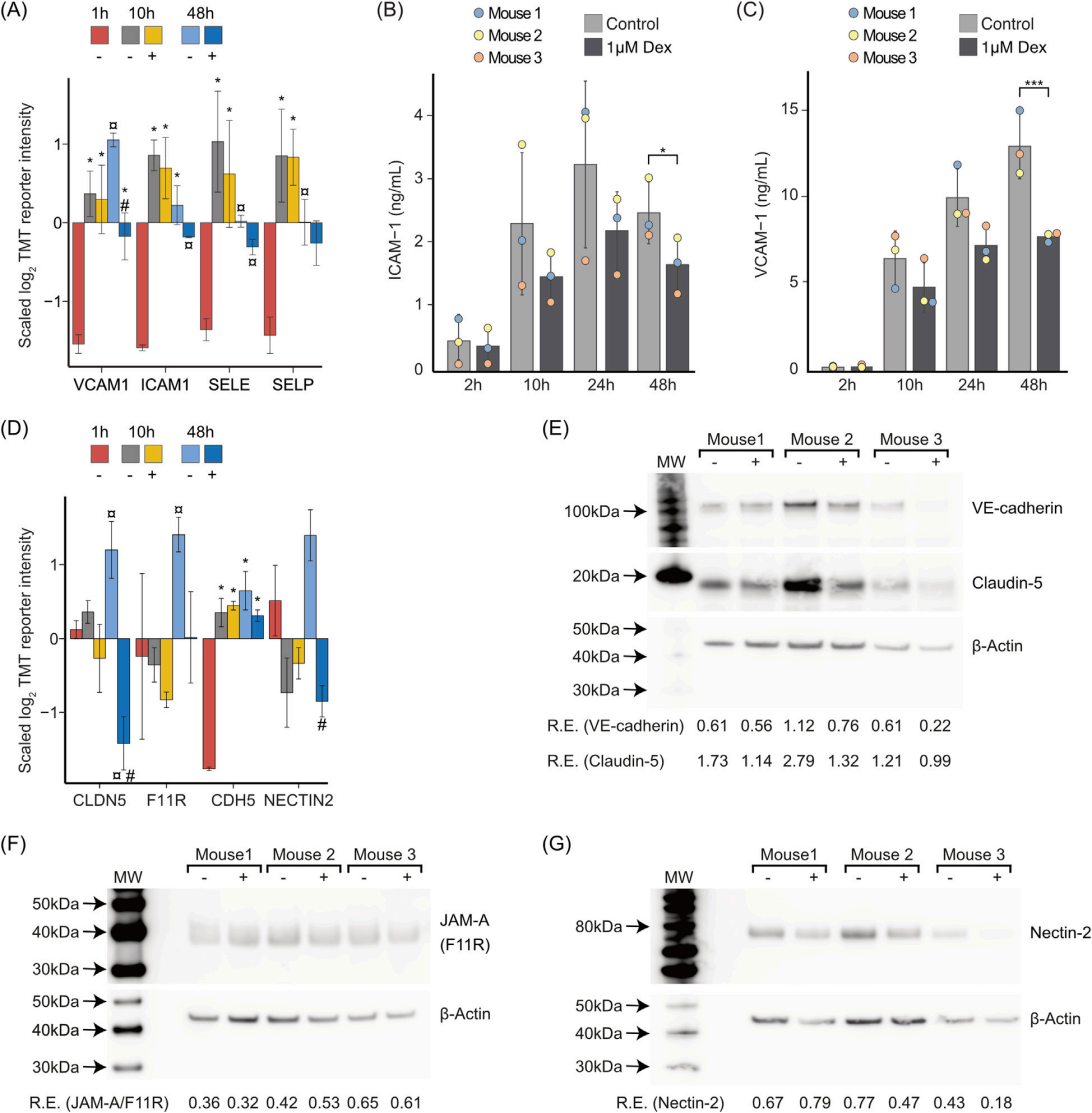
*In vitro* changes in cell adhesion molecules and junctional proteins, and effects of Dex. **(A)** Bar chart illustrating the average z-scaled log2TMT reporter intensities of cell adhesion molecules that are considered as biomarkers of endothelial activation (Zhang et al., 2010). The symbol *over the bar indicates significantly altered expression compared to 1 h; ¤ indicates significantly altered expression at 48 h compared to both 1 h and 10 h with or without Dex; # indicates significantly altered with Dex (+) compared to time-matched control (−). Proteins with |log2FC| ≥ 0.5 and FDR ≤ 0.05 in a pairwise comparison using an exact test in edgeR were identified as differentially expressed. Error bars show SD. **(B)** Concentration of ICAM-1 in LSEC culture supernatants at 2, 10, 24, or 48 h ± Dex (1 μM), measured by end-point ELISA. Results are mean values of 3 biological replicates ± SD. *p-value < 0.05 (pairwise t-test). **(C)** Concentration of VCAM-1 in LSEC culture supernatants at 2, 10, 24, or 48 h ± Dex (1 μM), measured by end-point ELISA. Results are mean values of 3 biological replicates ± SD. *p-value < 0.05 (pairwise t-test). **(D)** Bar chart showing tight junction proteins (CLDN5, F11 R), and adherens junction proteins (CDH5, NECTIN2) that were altered by time and/or Dex treatment. The symbol *over the bar indicates significantly altered expression compared to 1 h; ¤ indicates significantly altered expression at 48 h compared to both 1 h and 10 h with or without Dex; # indicates significantly altered with Dex (+) compared to time-matched control (−). Proteins with |log2FC| ≥ 0.5 and FDR ≤ 0.05 in a pairwise comparison using an exact test in edgeR were identified as differentially expressed. Error bars show SD. The underlying data for **(A)** and **(D)** is in Supplementary Table S4. **(E–G)** Western blots showing LSEC expression of **(E)** VE-cadherin (CDH5), claudin-5 (CLDN5), **(F)** F11R/JAM-A, and **(G)** nectin-2 (NECTIN2). Samples from 3 experiments, representing 3 individual mice were included in each blot. Digital scores show expression relative (R.E.) to the respective beta-actin control.

The GSEA (Figure 4C) showed significant enrichment of the term Cell adhesion molecules (CAMs). We found that ICAM-1 (ICAM1), VCAM-1 (VCAM1), E-selectin (SELE), and P-selectin (SELP) were enhanced both at 10 and 48 h. Of these, VCAM-1 was significantly repressed with Dex at 48 h (Figure 6A, Supplementary Table S4).

Increased expression of ICAM-1 and VCAM-1, both cell-bound and soluble, is reported biomarkers for endothelial activation (Ridker et al., 1998; Li et al., 1993). To quantitate the release of these proteins from LSEC cultures, we measured ICAM-1 and VCAM-1 in supernatants of cells incubated for 2, 10, 24, or 48 h in the presence or absence of Dex, in ELISA experiments. This showed that Dex repressed the release of both proteins (Figures 6B, C).

The analyses also revealed alterations in cell junction-associated proteins in culture, and counteracting effects of Dex (Figure 6D, Supplementary Table S4). Notably, LSECs (Sprague Dawley rat) are reported to lack the typical transmembrane tight junctional protein claudin-5 (CLDN5) and form special mixed-type intercellular junctions with tight junction and adherens junction proteins (Geraud et al., 2012). In our study, claudin-5 was significantly enhanced at 48 h (vs. 1 h control) in the absence of Dex whilst significantly suppressed in its presence (Figure 6D). Another tight junction protein, junctional adhesion molecule A (F11R, JAM-A) was also enhanced at 48 h in the non-treated cultures but not in the cultures with Dex (Figure 6D). The adherens junction protein cadherin-5 (CDH5, VE-cadherin) was enhanced in cultures both with and without Dex, with a slightly lower expression (not significant) in the Dex-treated cells at 48 h. Dex further showed a repressive effect on the enhancement of nectin cell adhesion molecule 2 (NECTIN2) at this time point (Figure 6D). We also examined the effect of Dex on the same proteins in western blot experiments, comparing protein expression in cells after 48 h with and without Dex. This revealed a similar trend as observed in the proteomic experiments for claudin-5, VE-cadherin, and nectin-2, showing a repressive effect of Dex (Figures 6E, G), while the expression of JAM-A/F11R was similar in the two groups (Figure 6F).

### 3.5 The changes in the LSEC proteome *in vitro* reflect a scenario of limited bioavailability of NO and redox imbalance

**FIGURE 7:**
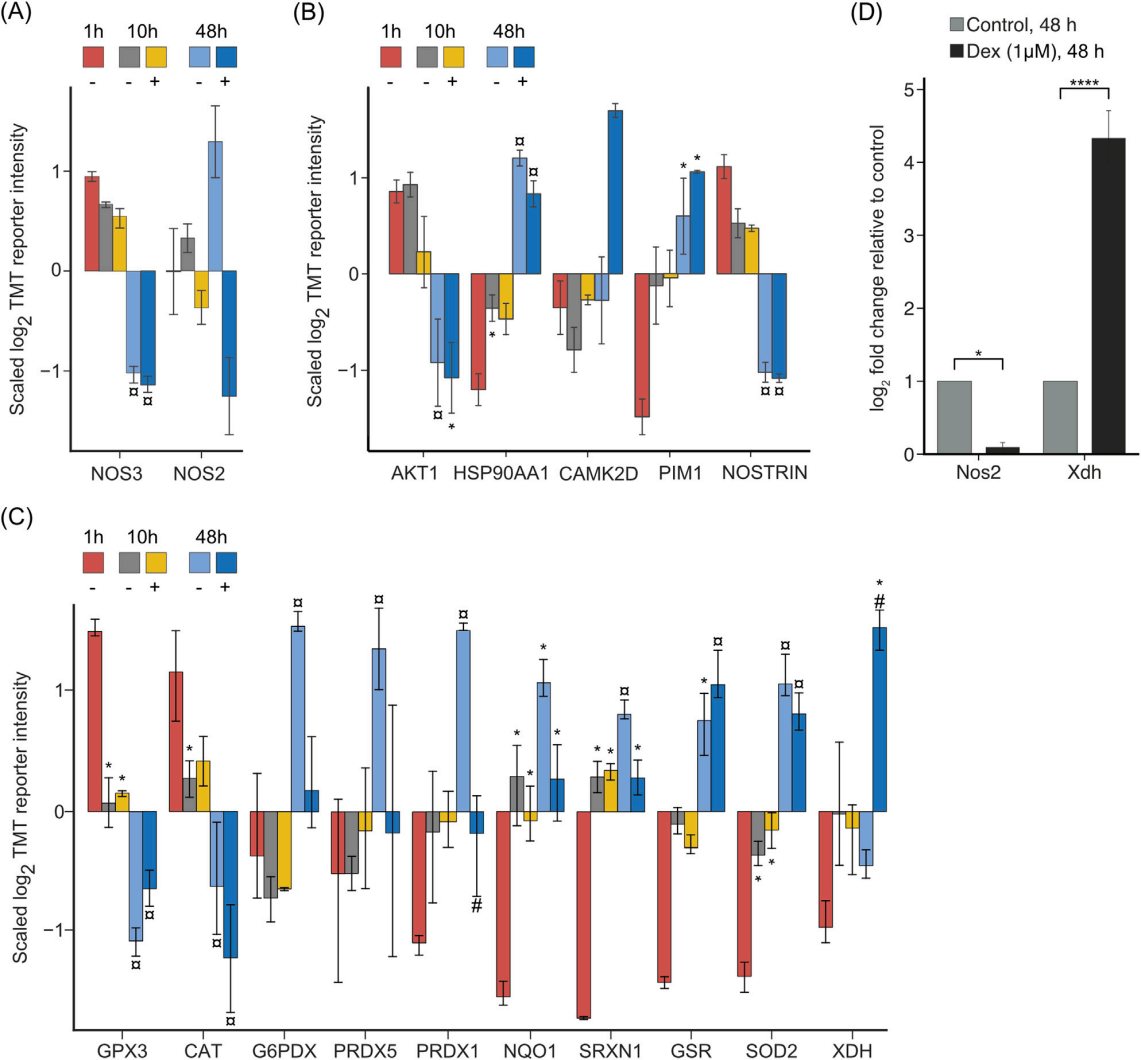
Proteins involved in NO production, and redox systems in LSECs. The bar charts in **(A–C)** illustrate the average z-scaled log2TMT reporter intensities of **(A)** the two isoforms of nitric oxide synthase, NOS2, and NOS3; **(B)** proteins involved in posttranslational modification of NOS3; and **(C)** selected proteins involved in the cell redox balance. In all figures * over the bar indicates significantly altered expression compared to 1 h; ¤ indicates significantly altered at 48 h compared to both 1 and 10 h with or without Dex; # indicates significantly altered with Dex (+) compared to time-matched control (−). Proteins with |log2FC| ≥ 0.5 and FDR ≤ 0.05 in a pairwise comparison using an exact test in edgeR were identified as differentially expressed. The underlying data for **(A–C)** is in Supplementary Table S4. **(D)** Effect of Dex on mRNA expression of Nos2 and Xdh in LSEC, analyzed by qPCR. Cells were treated for 48 h ± Dex. Results are presented as mean ± SD (biological replicates, n = 6) of expression related to the expression in non-treated control cultures, after first normalizing all expression values to B2m. *p-value ≤ 0.05; ****p-value ≤ 0.0001 (Student t-test).

The study revealed alterations in the expression of nitric oxide synthases and the regulation of the redox system in LSECs *in vitro*, both in the presence and absence of Dex (Figure 7, Supplementary Table S4). Nitric oxide synthase 3 (NOS3), also known as endothelial nitric oxide synthase (eNOS) was suppressed at 48 h, irrespective of Dex, whereas the expression of inducible nitric oxide synthase (NOS2, iNOS) at 48 h was enhanced without Dex and suppressed with Dex (Figure 7A). The effect of Dex on the expression of Nos2 was also measured by qPCR, validating the proteomic results (Figure 7D). Figure 7B shows changes in proteins affecting posttranslational modification, intracellular trafficking, and enzymatic activity of NOS3.

Differential protein expression analysis further showed derangement of the redox system in LSEC cultures (Figure 7C). Glutathione peroxidase 3 (GPX3) and catalase (CAT) were significantly downregulated both with and without Dex, glucose-6-phosphate dehydrogenase X-linked (G6PDX), and peroxiredoxin-1, and -5 (PRDX1, PRDX5) were upregulated only without Dex, whereas NAD(P)H: quinone oxidoreductase-1 (NQO1), sulfiredoxin-1 (SRXN1), glutathione-disulfide reductase (GSR), and superoxide dismutase 2 (SOD2) were upregulated both with and without Dex. Interestingly, xanthine dehydrogenase/oxidase (XDH), which is involved in the normal breakdown of purines but can also be involved in the production of superoxide radicals (Nishino et al., 2008) was significantly upregulated only by Dex (48 h) (Figure 7C). Upregulation of Xdh by Dex was validated in qPCR experiments (Figure 7D).

### 3.6 Dex inhibits apoptosis in LSECs *in vitro*

**FIGURE 8:**
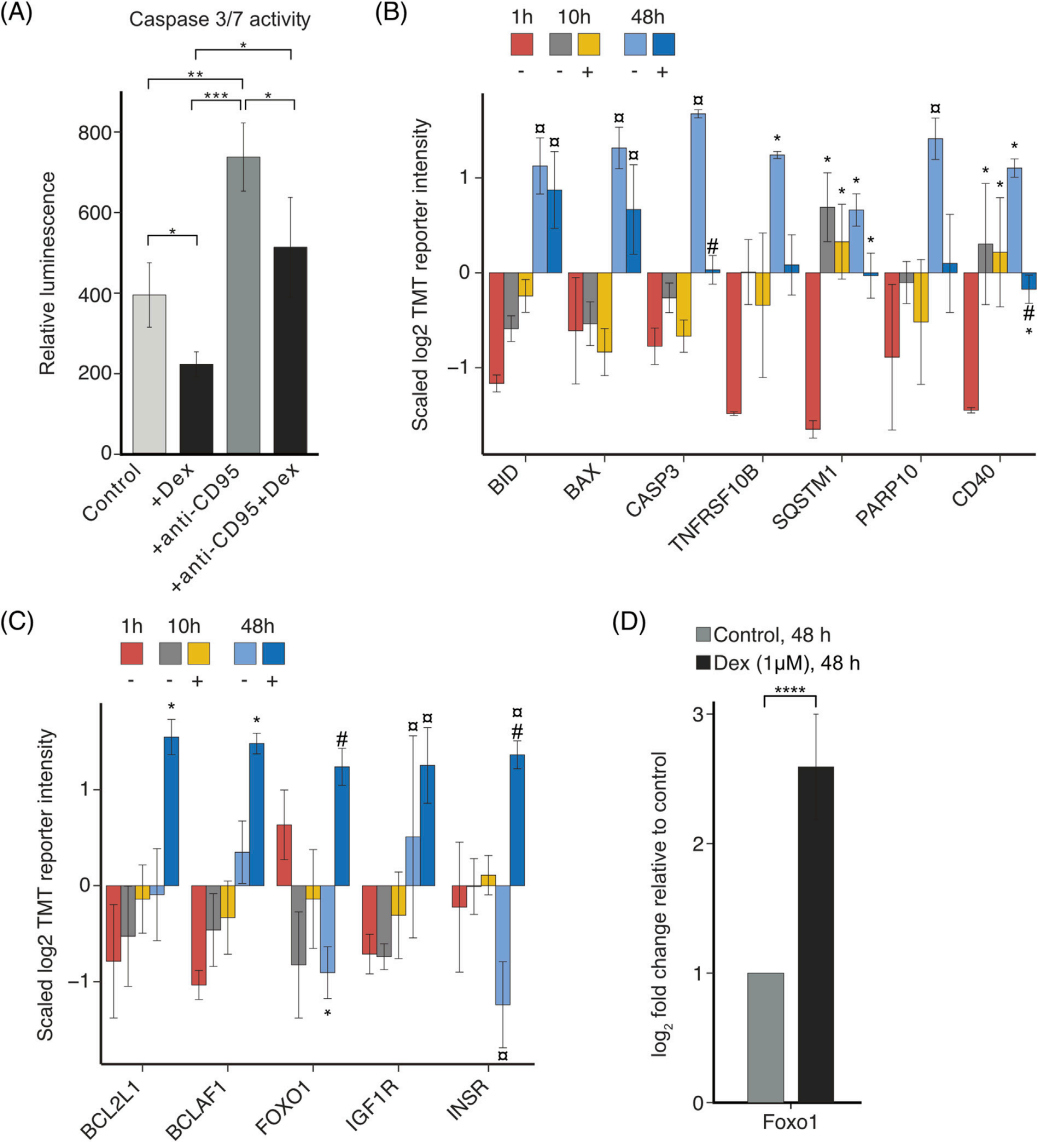
Dex effects on apoptosis in LSEC culture. **(A)** Bar chart illustrating changes in the relative luminescence unit (RLU), corresponding to apoptotic cell death in mouse LSECs cultured for 24 h without treatment, or with 1 μM Dex, anti-CD95, or 1 μM Dex + anti-CD95. Results are mean values of 3 biological replicates ± SD; * *p* < 0.05, ** < 0.01, ***<0.001 (ANOVA for dependent data, with Tukey´s *post hoc* test). **(B–C)** Bar charts illustrating the average z-scaled log2TMT reporter intensities of **(B)** pro-apoptotic proteins involved in the induction of cell death; **(C)** anti-apoptotic proteins providing a protective effect against cell death. The *over the bar indicates significantly altered expression compared to 1 h; ¤ indicates significantly altered expression at 48 h compared to both 1 and 10 h with or without Dex; # indicates significantly altered with Dex (+) compared to time-matched control (−). Proteins with |log2FC| ≥ 0.5 and FDR ≤ 0.05 in a pairwise comparison using an exact test in edgeR were identified as differentially expressed. Error bars show SD. The underlying data for **(B, C)** is in Supplementary Table S4. **(D)** Effect of Dex on mRNA expression of Foxo1 in LSECs, analyzed by qPCR. Cells were treated for 48 h ± Dex. Results are presented as mean ± SD (biological replicates, n = 6) of expression related to the expression in non-treated control cultures, after first normalizing expression values to B2m.****p-value ≤ 0.0001 (Student t-test).

Initial experiments demonstrated that Dex positively influenced LSEC viability in culture (Figure 2). To explore the mechanism in more detail, the impact of Dex on apoptosis was examined using a caspase 3/7 activity assay (Figure 8A). Results indicated that apoptosis was significantly lower in the Dex-treated cultures compared to the non-treated control cultures. Additionally, Dex significantly repressed the apoptosis stimulating effect of an anti-CD95 (FAS) antibody at 24 h (Figure 8A). Proteomics data revealed a significant upregulation of proapoptotic proteins at 48 h; these were partially repressed by Dex (Figure 8B, Supplementary Table S4). These proteins included BH3-interacting domain death agonist (BID), the apoptosis regulator BAX (BAX), caspase 3 (CASP3), tumor necrosis factor receptor superfamily member 10B (TNFRSF10B, also known as death receptor 5), sequestosome-1 (SQSTM1, p62), protein mono-ADP-ribosyltransferase (PARP10), and CD40. On the contrary, Dex supplementation enhanced the expression of Bcl-2-like protein 1 (BCL2L1, BCL-XL), which protects against BAX-induced apoptosis by sequestering BAX in a complex (Loo et al., 2020) (Figure 8C, Supplementary Table S4). Dex also stimulated the expression of the transcriptional repressor Bcl-2-associated transcription factor 1 (BCLAF1) which, when co-expressed with BCL2L1/BCL-XL, may counteract the apoptotic effect of BCLAF1 (Kasof et al., 1999). Dex further stimulated LSECs to induce the expression of proteins reported to enhance cell survival, including forkhead box protein O1 (FOXO1) (Greer and Brunet, 2005; Burgering and Medema, 2003), insulin-like growth factor 1 receptor (IGF1R), and insulin receptor (INSR) (Heidegger et al., 2014) (Figure 8C). The effect of Dex on Foxo1 expression in LSECs was further examined by qPCR, which showed significant upregulation of this transcription regulator by Dex, compared to the time-matched control (Figure 8D).

### 3.7 Dex did not rescue the *in vitro* decline in LSEC endocytic capacity

A hallmark function of LSECs is the high rate of clathrin-mediated endocytosis via a distinct set of scavenger receptors and C-type lectins (Bhandari et al., 2021; Pandey et al., 2020).

**FIGURE 9:**
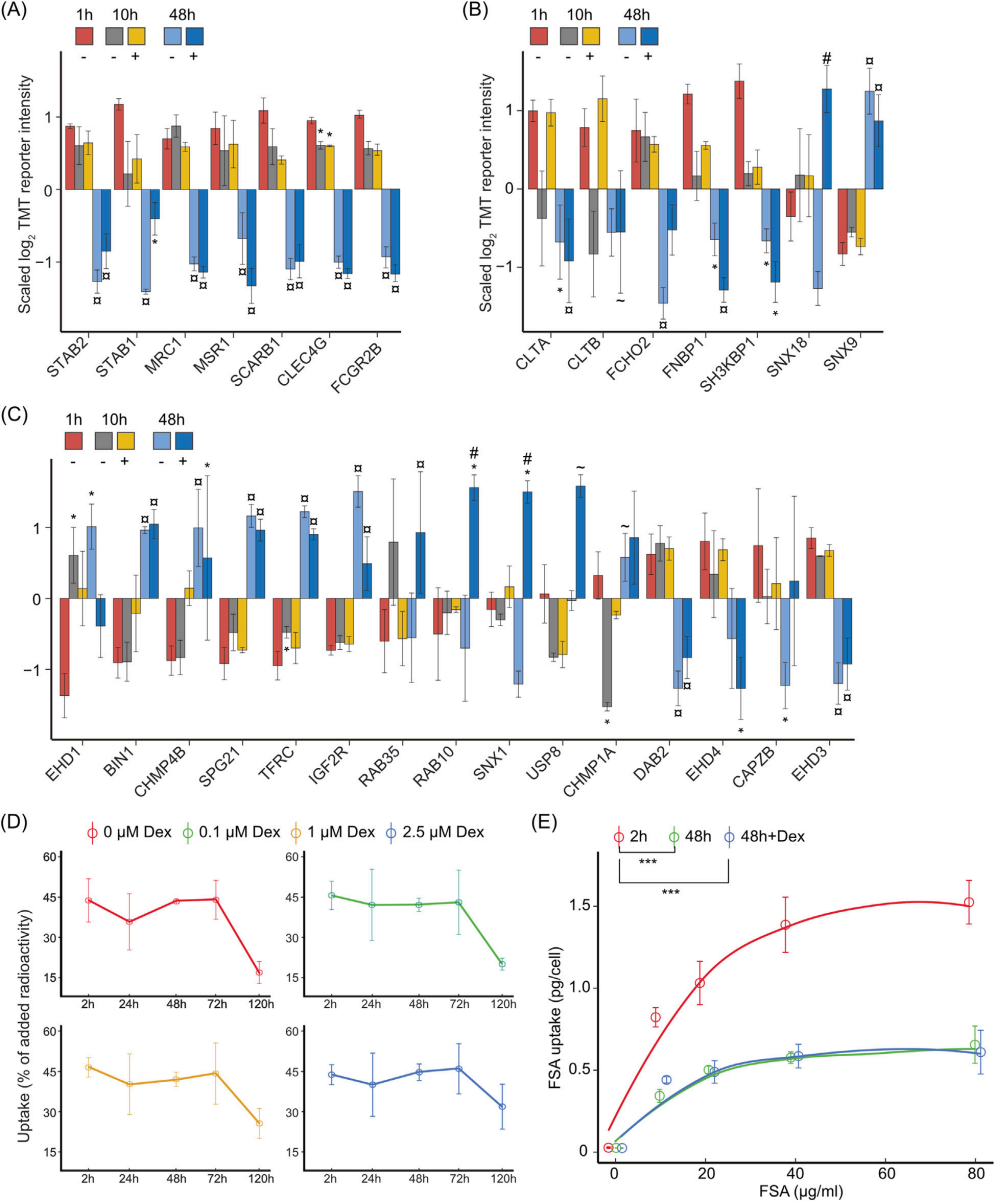
Dex effect on LSEC endocytosis function *in vitro*. The bar charts in **(A–C)** illustrate the average z-scaled log2TMT reporter intensities of **(A)** LSEC signature endocytosis receptors; **(B)** proteins associated with clathrin coat formation and vesicle maturation, and **(C)** proteins associated with endocytosis regulation in LSECs cultured for 1, 10, or 48 h ± Dex. The * over the bar indicates significantly altered expression compared to 1 h; ¤ indicates significantly altered expression at 48 h compared to both 1 and 10 h with or without Dex; # indicates significantly altered with Dex (+) compared to time-matched control (−); ∼ indicates significant changes between 48 h vs. 10 h compared with treatment-matched samples. Proteins with |log2FC| ≥ 0.5 and FDR ≤ 0.05 in a pairwise comparison using an exact test in edgeR were identified as differentially expressed. Error bars show SD. The underlying data for **(A–C)** is in Supplementary Table S4. **(D)** Endocytosis of low dose of formaldehyde-treated serum albumin (FSA) in LSECs. LSECs were kept in AIM-V medium ± Dex for 2, 24, 48, 72, or 120 h after culture establishment, and then incubated with ^125^I-FSA (approximately 0.1 μg/mL) for 2 h at 37°C. Endocytosis was measured as described in Methods, and results are given in percent of added radioactivity (±SD). **(E)** Capacity of mouse LSECs for endocytosis of FSA. Endocytosis was measured in freshly plated (2 h) LSECs, and in LSECs that had been cultured for 48 h ± 1 μM Dex before the start of the experiment. The cultures were then incubated with ^125^I-FSA (approximately 0.1 μg/mL) alone or together with nonlabelled FSA (10–80 μg/mL) for 2 h at 37°C, and the uptake per cell calculated as described in Methods. Error bars show SD (n = 3 biological replicates). Significant changes in endocytic capacity were determined by ANOVA for dependent data, followed by Tukey’s post-hoc test. *** *p* < 0.001.

Several LSEC signature receptors were significantly downregulated at 48 h, irrespective of Dex. This included stabilin-1 (STAB1), stabilin-2 (STAB2), mannose receptor C-type 1 (MRC1), macrophage scavenger receptor 1 (MSR1), scavenger receptor class B member 1 (SCARB1), C-type lectin domain family 4 member G (CLEC4G, LSECtin), and Fc fragment of IgG receptor IIb (FCGR2B) (Figure 9A, Supplementary Table S4).

We further found a time-dependent downregulation of the major coat proteins clathrin light chain A and B (CLTA, CLTB) as well as easy-arriving proteins that function in the early step of clathrin-mediated endocytosis [*i.e.*, F-bar domain only protein 2 (FCHO2), formin-binding protein 17 (FNBP1, FBP17), and SH3 domain-containing kinase-binding protein 1 (SH3KBP1), (Mettlen et al., 2018)] (Figure 9B, Supplementary Table S4). Exceptions were sorting nexin-18 (SNX18) which was enhanced by Dex at 48 h, and SNX9 which was enhanced at 48 h irrespective of Dex. Sorting nexins are multifunctional proteins and SNX9 and SNX18 are involved in clathrin-coated pit maturation and fission (Kaksonen and Roux, 2018).

Other essential components and regulators of endocytosis and vesicle trafficking were either up- or downregulated in culture (Figure 9C, Supplementary Table S4). Several proteins that were enhanced in culture are associated with membrane deformation and tubulation (BIN1, CHMP4B, SPG21) (van Weering et al., 2010), whereas proteins that were suppressed (DAB2, CAPZB, EHD3, EHD4) are associated with endocytosis receptor recycling. Interestingly, we found that SNX1 which is involved in endocytosis receptor recycling, and the hydrolase USP8 that deubiquitinates endocytosis-associated proteins which prevents lysosomal degradation (Grant and Donaldson, 2009; MacDonald et al., 2014), were enhanced in the presence of Dex, suggesting that Dex may modulate endocytosis in LSECs to some extent.

To investigate if/how the endocytic function of LSECs was affected by the observed changes in receptor expression and endocytic machinery, we examined the cellular uptake of formaldehyde-treated serum albumin (FSA), which is a commonly used test ligand for scavenger-receptor mediated endocytosis in LSECs (Sørensen et al., 2015), and bind to stabilin-1 and stabilin-2 in the cells (McCourt et al., 1999; Li et al., 2011). First, we examined for how long mouse LSECs in culture retained the ability to endocytose FSA. For this, we measured the uptake of a low dose of ^125^I-FSA (appr. 10 ng added per culture) during a 2 h incubation period in 0–5 days old cultures that had been treated with 0, 0.1, 1, or 2.5 μM Dex (Figure 9D). This showed that the uptake of ^125^I-FSA was highly efficient until 72 h post-seeding in all groups (approx. 45% of the ligand that was added to the culture where endocytosed by the cells in 2 h), and then dropped at 120 h (5 days) post-seeding, in a Dex dose-dependent manner.

Notably, this assay does not measure the maximum cell capacity of endocytosis, which may be reduced despite preserved rate of uptake of low ligand doses (Simon-Santamaria et al., 2010; Li et al., 2022). We therefore performed a different experiment to test the capacity of uptake of FSA per cell at 48 h post-seeding in cultures incubated in the presence or absence of 1 μM Dex. In these experiments, non-labeled FSA (10–80 μg/mL) was added to the culture in addition to the ^125^I-FSA tracer and results compared with the endocytic capacity of freshly plated LSECs. This showed that the endocytic capacity per cell was significantly reduced in LSECs that had been cultured for 48 h (Figure 9E), with no difference observed between Dex-treated and non-treated cells.

### 3.8 Time-dependent changes in transcriptional regulators in LSEC cultures, and effect of Dex

The proteomics study enlisted several transcriptional regulators (TRs) that may impact the LSEC phenotype *in vitro*. In total, we identified and cataloged 349 TRs out of 1,346 TRs listed in the databases Mouse Genome Informatics (MGI) (Bult et al., 2019), mouse tissue transcription factor atlas (Zhou et al., 2017), and Cistrome DB (Qin et al., 2020). Of these, the expression of 103 TRs was changed in a time-dependent or Dex-specific manner in the mouse LSEC proteome.

**FIGURE 10:**
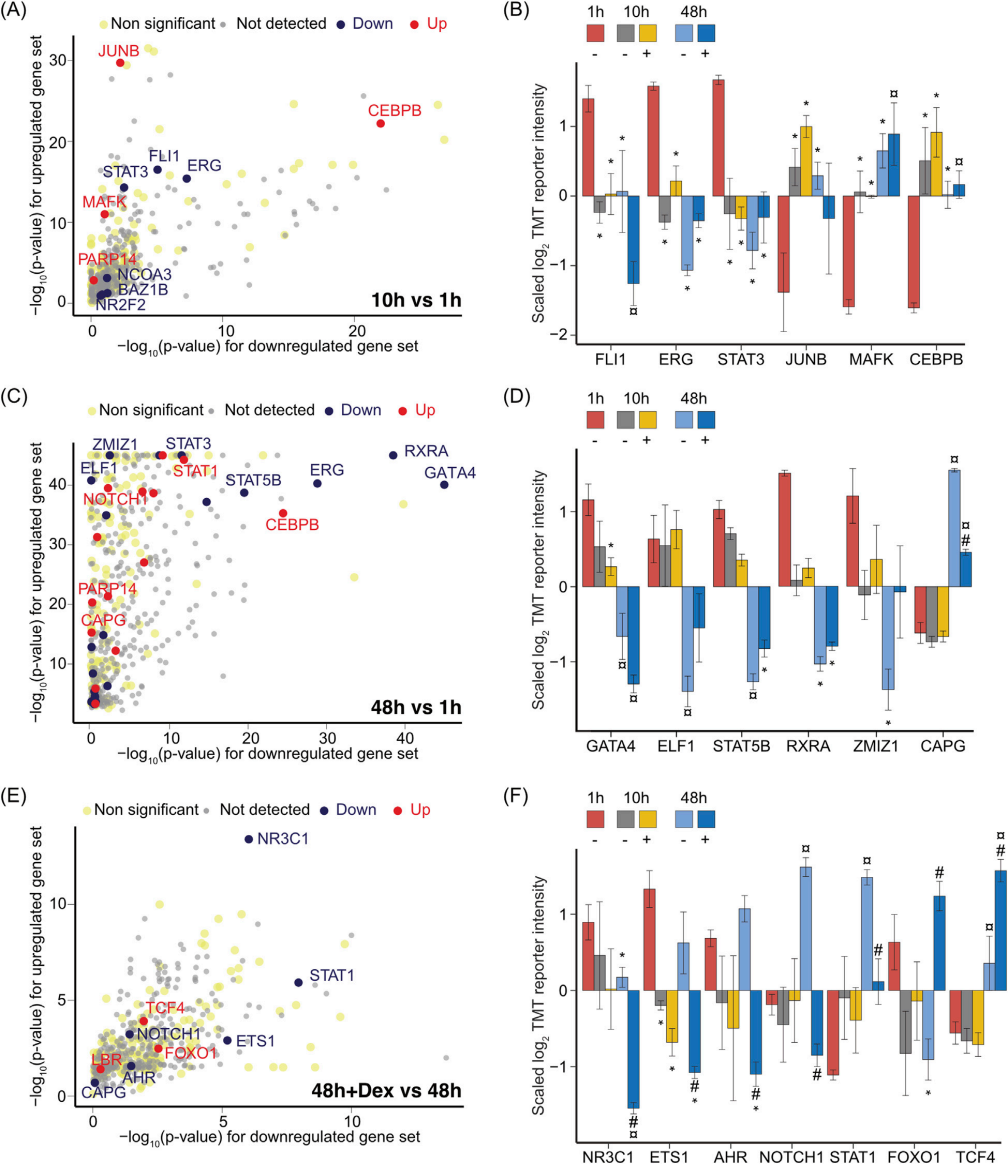
Effect of time in culture and Dex on LSEC transcriptional regulators. **(A)** Scatter plot showing the transcriptional regulators predicted by Lisa using the Cistrome DB (69), corresponding to up- or downregulated gene sets identified as significantly different between 10 h non-Dex LSEC samples vs. the 1 h non-Dex LSEC samples. Proteins with |log2FC| ≥ 0.5 and FDR ≤ 0.05 in a pairwise comparison using an exact test in edgeR were identified as differentially expressed. Each solid sphere indicates a transcriptional regulator in Cistrome DB. The grey spheres represent predicted proteins that were not detected in our TMT datasets. The light-yellow spheres were below the significant threshold of |log2FC| ≥ 0.5 and FDR ≤ 0.05. The black spheres represent transcriptional regulators that were significantly downregulated in LSECs at 10 h compared to 1 h, whereas the red-colored spheres show the ones that were significantly upregulated at 10 h. **(B)** Bar chart illustrating the average z-scaled log2TMT reporter intensities of transcriptional regulators that were significantly changed at 10 h *in vitro* in the LSEC proteome, predicted by the Lisa model (in **(A)**) based on the list of differentially expressed proteins between 10 h non-Dex samples vs. 1 h. Error bars show SD. **(C)** Scatter plot showing transcriptional regulators predicted by Lisa using the Cistrome DB, corresponding to up- or downregulated gene sets identified as significantly different between 48 h non-Dex samples vs. 1 h non-Dex samples. Proteins with |log2FC| ≥ 0.5 and FDR ≤ 0.05 in a pairwise comparison using an exact test in edgeR were identified as differentially expressed. Each solid sphere indicates a transcriptional regulator in Cistrome DB. **(D)** Bar chart illustrating the average z-scaled log2TMT reporter intensities of transcriptional regulators significantly changed at 48 h *in vitro* in the LSEC proteome, predicted by the Lisa model (in **(C)**) based on the list of differentially expressed proteins between 48 h vs. 1 h non-Dex treated samples. Error bars show SD. **(E)** Scatter plot showing the transcriptional regulators predicted by Lisa using the Cistrome DB, corresponding to up- and downregulated gene sets identified as significantly different between 48 h Dex-treated samples against 48 h non-treated samples. Proteins with |logFC| ≥ 0.5 and FDR ≤ 0.05 in a pairwise comparison using an exact test in edgeR were identified as differentially expressed. Each solid sphere indicates a transcriptional regulator in the Cistrome DB. **(F)** Bar chart illustrating the averages of the standardized log2TMT reporter intensities of transcriptional factors significantly changed at 48 h *in vitro* in a Dex specific manner in the LSEC proteome, predicted by the Lisa model (in E) based on the list of differentially expressed proteins between Dex-treated samples at 48 h vs. 48 h non-treated samples. Error bars show SD. Symbols in B, D, (**F)** Dex treatment is indicated by ‘+’ and no treatment by ‘–’. The * over the bar indicates significantly altered expression compared to 1 h; ¤ indicates significantly altered expression at 48 h compared to both 1 and 10 h with or without Dex; # indicates significant changes in the presence of Dex (+) compared to its time-matched controls (−). Proteins with |log2FC| ≥ 0.5 and FDR ≤ 0.05 in a pairwise comparison using an exact test in edgeR were identified as differentially expressed. The underlying data for **(A–F)** is in Supplementary Table S5.

Additionally, we implemented Local Indicators of Spatial Association (Lisa) models based on CistromeDB TR ChiP-seq (Qin et al., 2020) to predict TRs from the list of differentially regulated proteins, including early changes (10 h vs. 1 h) (Figure 10A), and later changes (48 h vs. 1 h) in the absence of Dex (Figure 10C), or changes caused by Dex, comparing 48 h with Dex vs. 48 h without Dex (Figure 10E). This analysis predicts which TRs are likely to drive the changes in the proteome. The corresponding expression of TRs in the proteomics datasets is shown in Figures 10B, D, F. Supporting data are in Supplementary Table S5. Interestingly, the top predicted TRs from the Lisa models were also the ones that were significantly altered in the LSEC proteomes.

At 10 h, the expression of FLI1 (Friend leukemia integration 1 transcription factor), ERG (Transcriptional regulator ERG), and STAT3 were repressed, whereas JUNB (Transcription factor jun-B), MAFK (Transcription factor MafK), and CEBPB (CCAAT/enhancer-binding protein beta) were enhanced compared to 1 h (Figure 10B). Recently, a constitutive expression and cooperative action of FLI1 and ERG were shown essential to uphold endothelial identity, while deficiency of these factors leads to leakiness and increased inflammation (Gomez-Salinero et al., 2022).

At 48 h, GATA4 (transcription factor GATA-4), which is an essential transcription factor for the development and maintenance of the LSEC phenotype (Geraud et al., 2017), was repressed both in the presence and absence of Dex (Figure 10D). ERG, STAT3 (Figure 10B) ELF1 (ETS-related transcription factor Elf-1), STAT5B, RXRA (Retinoid X receptor alpha) and ZMIZ1 (Zinc Finger MIZ-type containing 1) (Figure 10D) were also repressed at this time point in culture, while LSECs displayed elevated expression of CAPG (Macrophage-capping protein) (Figure 10D), and STAT1 (Figure 10F). The observed changes in top predicted TFs at 10 and 48 h suggest that the LSEC phenotype undergo marked transcriptional reprogramming over time.

Significant differences in TR expression between Dex-treated cells and time-matched controls, were first observed in the proteome at 48 h. NR3C1, ETS1, AHR (Aryl hydrocarbon receptor), NOTCH1, STAT1, FOXO1, and TCF4 (Transcription factor 4), shown in Figure 10F were among the top 10 predicted TRs identified as regulated by Dex at this time point (Figure 10E). The glucocorticoid receptor NR3C1 was downregulated in the LSEC proteome, and significantly further repressed in the presence of Dex (Figure 10F). ETS1 (ETS proto-oncogene 1), which activates the expression of cytokine and chemokines in various contexts (Russell and Garrett-Sinha, 2010), was also repressed in the Dex-treated samples (Figure 10F). NOTCH1, a regulator of cell proliferation, differentiation, and cell death (Kopan, 2012), and STAT1, which is involved in immune system functions, were enhanced in the non-treated samples at 48 h, while Dex reciprocated or repressed this upregulation (Figure 10F). On the contrary, FOXO1 which is an important regulator in energy metabolism and activator of gluconeogenesis and glycogenolysis (Calissi et al., 2021) was repressed without Dex, and enhanced with Dex (Figures 10F, 8D).

## 4 Discussion

In this study, we have performed functional assays and generated a fine-grained snapshot of the mouse LSEC proteome at 1, 10, and 48 h post plating of cells freshly isolated from liver, with focus to uncover early changes in the LSEC proteome *in vitro,* and cell-specific responses to Dex treatment.

The proteomic enrichment analysis reflected an immediate, and substantial shift in the metabolism of mouse LSECs in culture, indicating increased glycolysis and diminished oxidative phosphorylation both in the presence and absence of Dex, with a modulating effect of Dex. A similar shift in metabolic pathways was observed in the proteome of primary rat LSECs in culture (Li et al., 2022).

It has been reported that rat LSECs likely generate 80% of their ATP from glutamine and palmitate oxidation *in vitro* (Spolarics et al., 1991). However, our proteomics data indicated that LSECs depend more on glycolysis for ATP regeneration, at least in medium with normal/high glucose level, such as AIM-V (used in the present study), and DMEM, used in (Li et al., 2022). This is consistent with previous reports which show a high production of lactate and acetate in primary LSECs *in vitro* (Smedsrød, 1991; Smedsrød et al., 1984; Falkowska-Hansen et al., 2006; Nedredal et al., 2009). In bioreactors with pig LSECs and hepatocytes, the LSEC reactor produced 5-fold more lactate/million cells than the hepatocytes (Nedredal et al., 2009). Interestingly, assessment of bioenergetics in LSECs isolated from mice fed on a high fat diet suggested that LSECs are energetically flexible. In the initial stages of fatty liver disease, the LSEC mitochondrial respiration was impaired and compensated by increased basal glycolysis, while at a later stage in the disease, this balance was changed in the favor of mitochondrial respiration (Kus et al., 2019).

A limitation of proteomics experiments is that they only provide information about protein expression levels. To get a full understanding of cell metabolism, functional experiments with specially designed media and assays need to be performed. However, our study clearly shows that the expression of proteins in metabolic pathways change fast in LSEC primary culture, confirming recent results in rat LSECs (Li et al., 2022). This represents a challenge when extrapolating results of metabolic studies performed in LSECs *in vitro* to the *in vivo* situation in liver.

A shift towards increased glycolysis has been reported to be characteristic of pro-inflammatory immune cells (Jha et al., 2015; Wculek et al., 2019), and activated non-LSEC endothelial cells (Kalucka et al., 2018). We found that primary mouse LSECs, like rat LSECs (Li et al., 2022) acquired an activated phenotype short time after plating, featured by elevated expression, and secretion, of pro-inflammatory proteins and cell adhesion molecules, which was reduced by Dex. We also observed a culture-induced enhancement of several cell junction-associated proteins (CLDN5, F11R, CDH5, and NECTIN2), which was partly or fully repressed by Dex compared to the time-matched controls at 48 h (validated in western blots for CLDN5, CDH5, and NECTIN2 but not for F11R). Interestingly, the scanning EM experiments revealed that LSECs cultured with Dex were more closely connected to each other, with less gaps between cells, compared to the cells in the non-treated, time-matched control cultures (Figure 3A). The observed changes in cell junction-associated proteins suggests that the *in vitro* conditions induced pathological alterations in LSEC junctional complexes, which were counteracted by Dex treatment. This opens for the hypothesis that Dex in the tested doses has a positive effect on the integrity of LSEC cell junctions.

The cell activation in culture may be caused by stress responses to the cell isolation procedure and non-physiological substrate (March et al., 2009; Juin et al., 2013; Denisenko et al., 2020; O’Flanagan et al., 2019). The increased expression of NOTCH1 at 48 h, which was reciprocated by Dex (Figure 10F), supports LSEC activation in culture. Derangement in angiocrine factors such as NOTCH1 is a feature of endothelial activation (Csiszar et al., 2008), and NOTCH1 activation has been shown to cause dedifferentiation and pro-inflammatory activation of LSECs in mouse studies (Zhang et al., 2020; Duan et al., 2018).

Limited bioavailability of nitric oxide (NO) is reported in the literature as a key factor contributing to the loss of LSEC function (DeLeve et al., 2003). NO signaling affects endothelial cell physiology and pathophysiology including metabolism, vascular tone, and immune responses (Oliveira-Paula et al., 2017). In the liver, NO production is catalyzed by endothelial nitric oxide synthase (NOS3; eNOS), and inducible NOS (NOS2; iNOS) (Iwakiri and Kim, 2015). We found that mouse LSECs in culture showed decreased expression of NOS3, while the expression of NOS2 was increased, suggesting a deranged redox system, supported by the proteomics data (Figure 7). Diminished expression of NOS3, combined with elevated expression of NOS2 and downregulation of AKT1, which was also observed in our study, is reported to favor uncoupling of NOS3 effects leading to the production of reactive oxygen species (Iwakiri and Kim, 2015). Dex suppressed the culture-induced increase in NOS2 but did not rescue downregulation of NOS3, consistent with the effect of Dex on NOS2 and NOS3 expression in rat LSECs (Li et al., 2022). NOS3 dependent NO release is hepatoprotective and maintains LSEC fenestration as well as promotes hepatic stellate cell and Kupffer cell quiescence (Iwakiri and Kim, 2015; Xie et al., 2012). NOS3 is upregulated in response to VEGF, shear stress (via the transcription factor KLF2), and transcriptional enhancers such as ELF1, EST1, and ERG (Oliveira-Paula et al., 2016) which were repressed in the mouse LSEC cultures in the presence and/or absence of Dex (Figure 10). Downregulation of NOS3 expression in our cell system may therefore be partly explained by lack of VEGF-signaling, and a static, monocellular culture system.

NOS2 is induced through activation of NF-κB and STAT-pathways and increases NO production in the cells in response to pro-inflammatory stimuli (Farlik et al., 2010). The downregulation of NOS2 in Dex-treated LSECs may therefore be a consequence of repression of NF-κB signaling pathways, as indicated by the GSEA (Figure 4C). NOS2-derived NO contributes to reactive nitrogen species that promotes inflammation (Iwakiri and Kim, 2015). In rat LSECs, an elevated level of NO in response to pro-inflammatory cytokine exposure was reported to downregulate endocytosis via scavenger- and mannose receptors (Martinez et al., 1996).

XDH (xanthine dehydrogenase/oxidase) was enhanced in LSEC cultures treated with Dex, both at protein and mRNA level compared to the time-matched control (Figures 7C, D). XDH uses NAD^+^ as an electron acceptor to convert hypoxanthine and xanthine into uric acid while producing NADH. This reaction supports cellular energy balance by contributing to the NAD^+^/NADH ratio (Pacher et al., 2006). However, from the present study we cannot state how the observed protein/mRNA changes in XDH affects the LSECs. XDH is part of xanthine oxidoreductase (XOR), which is an enzyme complex with versatile functions. Xanthine dehydrogenase in this complex can be converted to xanthine oxidase by post translational modification. The XOR complex is linked to oxidative stress and generates reactive oxygen species, and NO (Bortolotti et al., 2021). Notably, we have cultured LSEC in 5% O_2_ which is recommended for LSECs to minimize oxidative stress (Martinez et al., 2008), and XOR has been shown to catalyze production of NO from reduction of nitrate and nitrite under hypoxic conditions. *In vivo*, the generation of NO by XOR affects vascular tone and endothelial function by promoting vasodilation (Millar et al., 1998).

A significant effect of Dex on the LSEC cultures was improved cell viability and a positive effect on the cell morphology, with smoother cell borders, and less gaps between cells, and a slightly delayed defenestration of the cells compared to time-matched non-treated cells after 24–48 h. Beside repression of inflammatory pathways, the pro-survival effect of Dex on mouse LSECs *in vitro* may be partly explained by the repression of pro-apoptotic proteins (Figure 8B), combined with the enhanced expression of anti-apoptotic proteins and survival factors (Figure 8C). The anti-apoptotic effect of Dex on LSECs was validated in a caspase 3/7 activity assay. Notably, LSECs did not proliferate in cultures, irrespective of Dex (Supplementary Figure S1C).

Dex is commonly used as cell culture supplement and has a broad range of effects on cell viability, depending on cell type, and dose (Quatrini and Ugolini, 2021). In the liver, cumulative evidence from *in vitro* studies supports a positive effect of Dex on hepatocyte viability and even a low concentration (100 nM) inhibits primary hepatocytes from undergoing apoptosis in culture (Morin and Normand, 1986; Turncliff et al., 2004). In the clinic, Dex is used over a wide dose range both as an anti-inflammatory/immune modulatory drug, and in cancer treatment where pro-apoptotic effects are beneficial (Lin and Wang, 2016). In patients, high doses and prolonged treatment of Dex have several adverse side effects, like cushingoid changes (abdominal obesity, face swelling, easily bruised skin), increased risk for infections, glaucoma, and elevated blood pressure (Poetker and Reh, 2010). The present study did not reveal Dex toxicity in LSECs, even at high doses in the functional assays *in vitro*. However, we found that expression of FOXO1 and insulin receptor (INSR) were enhanced by Dex (1 μM) at 48 h compared to time-matched non-treated controls. FOXO transcription factors are targets of insulin signaling in cells and play an important role in regulating gene expression related to gluconeogenesis, glycolysis, and energy metabolism in the liver (O-Sullivan et al., 2015). FOXO1 is here involved in the regulation of insulin response, and fasting blood glucose rises when FOXO1 is increasingly expressed in the liver (Matsumoto et al., 2006; Tikhanovich et al., 2013). Glucocorticoids decrease the hepatic and systemic sensitivity to insulin and induce insulin resistance (Protzek et al., 2016). Insulin resistance can develop in endothelial cells (Muniyappa and Quon, 2007), and it may be hypothesized that Dex-mediated upregulation of Foxo1 expression in LSEC over time can lead to insulin resistance in these cells.

The anti-inflammatory action of Dex depends prominently on the nuclear receptor NR3C1. NR3C1 bound to Dex translocate into the nucleus to activate anti-inflammatory gene expression and represses NF-ĸB and AP-1 mediated pro-inflammatory gene expression (Smoak and Cidlowski, 2004). NR3C1 was downregulated in a time-dependent manner in the mouse LSEC cultures and NR3C1 repression was significantly more pronounced in the cells cultured with Dex in the proteome dataset. Dex-mediated degradation of NR3C1 has been shown in other cell models (Sengupta and Wasylyk, 2001; Silva et al., 1994; Wallace and Cidlowski, 2001; Wandler et al., 2020). Dex treatment for 48 h also resulted in the elevated level of the co-chaperone FKBP prolyl isomerase 5 (FKBP5; Supplementary Table S1), which inhibits NR3C1 translocation into the nucleus (Guidotti et al., 2013), suggesting that LSECs might lose sensitivity towards Dex in prolonged culture, as reported for other cell types (Guidotti et al., 2013; Mata-Greenwood et al., 2013).

Efficient clathrin-mediated endocytosis of soluble macromolecules via scavenger receptors is a hallmark of LSEC integrity (Martinez et al., 2008). FSA is commonly used as a model ligand for the study of scavenger receptor-mediated endocytosis in LSECs (Antwi et al., 2023; Sørensen et al., 2015; DeLeve and Maretti-Mira, 2017; Holte et al., 2023; Kyrrestad et al., 2023), and the uptake of FSA in LSECs is mediated via stabilin-1 (STAB1) and stabilin-2 (STAB2) (McCourt et al., 1999; Li et al., 2011). The LSEC endocytic function is known to decline in culture (Martinez et al., 2008; Li et al., 2022), and LSEC cell lines with preserved scavenger function are still not available (Poisson et al., 2017). We found that mouse LSECs in AIM-V medium preserved the ability to rapidly endocytose trace doses of radio-iodinated FSA for at least 5 days in culture. After 5 days this ability was best preserved in Dex-treated cultures. However, the maximum capacity for FSA uptake per cell was markedly reduced (measured at 48 h versus 2 h), with no significant improvement by Dex. In accordance with this observation, the proteomics data showed diminished expression at 48 h of central LSEC scavenger receptors and C-type lectins (STAB1, STAB2, MRC1, MSR1, SCARB1, CLEC4G, FCGR2), together with several proteins involved in clathrin-mediated coat formation and regulation of endocytosis, irrespective of Dex-treatment (Figure 9). Dex induced upregulation of sorting nexins involved in clathrin-mediated endocytosis and recycling, which may positively influence the rate of endocytosis (Figure 9D). However, the higher rate of uptake of trace doses of FSA in the 5 days cultures with Dex can be explained by the observed increased survival of Dex-treated cells.

GATA4 has been repeatedly shown to be a crucial transcription factor for the development and maintenance of the LSEC phenotype (Geraud et al., 2017; Geraud et al., 2010; de Haan et al., 2020; Olsavszky et al., 2017). This factor was significantly downregulated in the mouse LSEC proteome at 48 h, irrespective of Dex treatment. Downregulation of GATA4 was also reported in primary rat LSECs, and after 2 days in culture the cells had acquired a phenotype that resembled rat lung endothelial cells more closely compared to freshly isolated LSECs (Geraud et al., 2010). From a panel of 7 LSEC-specific transcription factors found by comparing endothelial cells from liver, heart, and brain, GATA4, in combination with MEIS2 (MEIS homeobox 2) and C-MAF (musculoaponeurotic fibrosarcoma) showed a strong synergistic effect on the induction of LSEC genes in human umbilical cord vein endothelial cells (de Haan et al., 2020).

In conclusion, we report the detailed changes in the mouse LSEC proteome during the first 10 h in culture, and after prolonged culture (48 h), and the net effect of Dex on the proteome, viability, and morphology of the cells. Dex is widely used in the clinic, and it is therefore important to know how the drug affects different cell types in the body. Additionally, a deep understanding of the proteome changes that occur in LSECs *in vitro* may support the work to improve LSEC culture systems and interpretation of *in vitro* studies in these cells. Early management of the inflammatory changes and sustaining the expression of the transcriptional regulators altered *in vitro* may improve LSEC culture systems. Our study further highlights the importance of monitoring culture-dependent (i.e., non-drug dependent) changes in primary culture when measuring drug effects on the cells.

## Data Availability

The original datasets are deposited in the ProteomeXchange repository, accession number PXD041381. The data is available at: https://proteomecentral.proteomexchange.org/ui?search=PXD041381 / the whole processed proteome is included in Supplementary Material.

## Supplementary material

SUPPLEMENTARY FIGURE S1 LSEC morphology after 5 days, and test of LSEC proliferation in culture. **(A)** Phase contrast images of LSEC cultures 5 days after plating cells freshly isolated from mouse liver. The cells were cultured in AIM-V medium ± Dex at indicated doses, in 5% O2, 5% CO2 atmosphere. NTC, non-treated control. **(B)** Scanning electron micrographs of non-treated (NTC) and Dex-treated (2.5 μM) 5 days old mouse LSEC cultures. **(C)** Cell proliferation assay (BrdU incorporation). Mouse embryonic fibroblasts (MEF) cells (blue circles) were used as positive control. Results for LSECs are indicated by orange circles. Biological replicates, n=3.

SUPPLEMENTARY TABLE S1 The Excel-file contains the whole processed proteomics dataset, and comparison of protein expression between groups.

SUPPLEMENTARY TABLE S2 The Excel-file contains the results of Gene Set Enrichment Analysis (GSEA) presented in Figure 4C. The analysis was based on hallmark gene sets and KEGG legacy pathway gene sets defined in the Molecular Signatures Database (MSigDB ver 7.2) (Subramanian et al., 2005).

SUPPLEMENTARY TABLE S3 The Excel-file contains the proteomics dataset for Figure 5.

SUPPLEMENTARY TABLE S4 The Excel-file contains the proteomics datasets for Figures 6–9.

SUPPLEMENTARY TABLE S5 The Excel-file contains the results of the LISA predictions presented in Figures 10A, C, E, and the proteomics datasets for Figures 10B, D, F.
