# Supplementary figures and images for: Mouse liver sinusoidal endothelial cell responses to the glucocorticoid receptor agonist dexamethasone
Source: Front Pharmacol. 2024 Oct 8;15:1377136. doi: 10.3389/fphar.2024.1377136 (PMC11494038; doi:10.3389/fphar.2024.1377136)

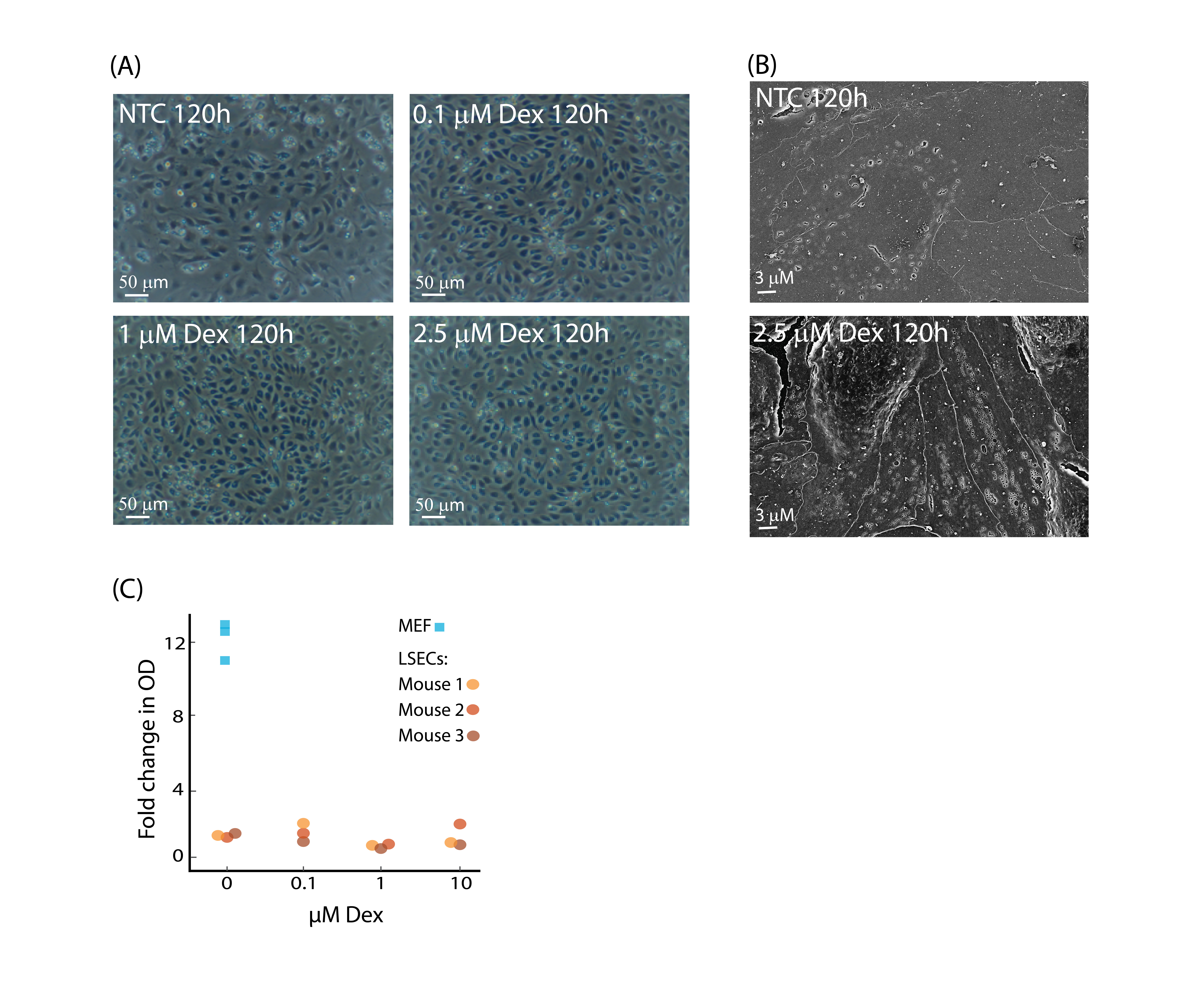

Supplement: Supplementary file 3 [file Image1.JPEG]
